# Lymphocyte subset reconstitution and clinical outcomes following haploidentical hematopoietic stem cell transplantation

**DOI:** 10.1038/s41416-026-03345-w

**Published:** 2026-03-02

**Authors:** Peiyao Jiang, Xiao Zhou, Yu Cai, Jun Yang, Kun Zhou, Yin Tong, Chongmei Huang, Baoxia Dong, Huiying Qiu, Xiaowei Xu, Jiahua Niu, Chang Shen, Xinxin Xia, Ying Zhang, Xianmin Song, Liping Wan

**Affiliations:** Department of Hematology, Shanghai General Hospital, Shanghai Jiao Tong University School of Medicine, Shanghai, China

**Keywords:** Haematological cancer, Lymphocytes, Haematological cancer

## Abstract

**Background:**

Immune reconstitution is critical for outcomes after allogeneic hematopoietic stem cell transplantation (allo-HSCT), but its prognostic role in haploidentical peripheral blood stem cell transplantation (haplo-PBSCT) remains unclear.

**Methods:**

We retrospectively analysed 577 patients who underwent T-cell replete haplo-PBSCT between 2016 and 2024. Longitudinal reconstitution of CD4⁺, CD8⁺ T cells and their subsets, CD3⁺CD69⁺, CD3⁺HLA-DR⁺ T cells, NK cells and B cells was assessed from 1 to 12 months post-transplant. Cox regression and causal mediation analyses were performed to identify prognostic associations and mechanisms.

**Results:**

Early and robust regulatory T cell (Treg) reconstitution significantly reduced transplant-related mortality (TRM) and improved overall survival. Naïve CD8⁺ T cell recovery correlated with reduced TRM and relapse. Higher late CD3⁺CD69⁺ T cells were linked to decreased relapse risk. Sustained B cell reconstitution reduced TRM and was associated with a lower incidence of moderate-to-severe chronic graft-versus-host disease. Early Treg reconstitution was associated with lower cytomegalovirus (CMV) reactivation. Mediation analysis revealed that Treg recovery reduced TRM partially through suppression of CMV reactivation (ACME = –0.22, *p* = 0.032).

**Conclusion:**

Distinct lymphocyte subset reconstitution profiles predict transplant outcomes in haplo-PBSCT. Early Treg recovery, partly by limiting CMV reactivation, may serve as a target for immune-guided intervention.

## Introduction

Allogeneic hematopoietic stem cell transplantation (allo-HSCT) is a curative treatment modality for most haematological malignancies. Recently, haploidentical HSCT has become widely utilised, providing critical therapeutic options for patients lacking HLA-matched donors. The efficacy of allo-HSCT is primarily mediated by graft-versus-tumour effects [1–3]. Successful immune reconstitution following HSCT significantly reduces infection and relapse risk, and consequently improving survival outcomes [4, 5]. Some studies have demonstrated improved overall survival (OS) and reduced transplant-related mortality (TRM) associated with rapid and robust lymphocyte recovery post-transplant in the HLA-matched setting [6–10]. Buhlmann et al. reported that early reconstitution of natural killer (NK) cells and CD4^+^ T cells independently correlated with reduced TRM in patients receiving T-cell replete HLA-identical transplants [9]. Similarly, Minculescu et al. demonstrated that NK cell reconstitution by day 30 post-transplantation independently predicted improved OS following HLA-identical HSCT [10]. Additionally, data from a retrospective study indicated that CD8^+^ T cell count above 50 × 10^6^ cells/L on day 28 post-transplant was associated with a lower risk of relapse, while lower counts correlated with increased relapse frequency [11]. Roessle et al. reported that early CD4^+^ T cell immune reconstitution was associated with superior OS and event-free survival, although no significant impact on relapse was noted [12]. Furthermore, another recent study suggested that achieving CD4^+^ T cells >50 cells/µL or B cells >25 cells/µL before day 100 post-transplant predicted lower TRM and relapse risks in patients with acute myeloid leukaemia (AML) [13].

However, previous studies primarily focused on the impact of early immune reconstitution on survival, without comprehensively evaluating the long-term reconstitution trajectories of specific lymphocyte subsets and their distinct effects on clinical outcomes. Moreover, the relationship between immune reconstitution kinetics and clinical outcomes in haploidentical HSCT remains inadequately defined. Critical questions remain unanswered in haploidentical transplantation, particularly regarding which lymphocyte subsets exert the most significant impact on transplant outcomes and at which specific post-transplant time points their reconstitution most effectively influences TRM and relapse.

To address these knowledge gaps in a biologically informed way, we profiled T-cell compartments (CD4⁺ and CD8⁺ with their naïve [CD45RA⁺] and memory [CD45RO⁺] subsets), regulatory T cells (Tregs, CD4⁺CD25⁺CD127^–^), early- and late-activated T cells (CD3⁺CD69⁺ and CD3⁺HLA-DR⁺), NK cells and B cells. These populations capture complementary axes of post-transplant immunity—regulation vs. effector function (Tregs vs. conventional T cells), maturation state (naïve vs. memory), activation status (CD69/HLA-DR), and innate/humoral compartments (NK/B cells). Prior allo-HSCT work links inadequate Treg activity to GVHD and loss of immune homoeostasis [14–16], early NK reconstitution to favourable survival [9, 10] and impaired B-cell recovery to infection susceptibility [17]. Naïve T-cell recovery also reflects thymic output—central to long-term immune competence—whereas activation markers index recent T-cell activation and may relate to graft-versus-leukaemia activity. Therefore, we sampled at 1, 2, 3, 4, 6, 9 and 12 months post-transplant in a large cohort of patients undergoing T-cell–replete haploidentical peripheral blood stem cell transplantation (haplo-PBSCT) and we retrospectively analysed the reconstitution dynamics of multiple lymphocyte subsets. Our study demonstrated a clear association between the reconstitution kinetics of specific lymphocyte subsets—particularly Tregs and B cells—and clinical outcomes, thereby highlighting their prognostic significance in the context of haploidentical transplantation.

## Methods

### Patients and donors

A total of 662 patients with haematological malignancies who underwent haplo-PBSCT at our institution between March 2016 and January 2024 were initially screened in this study. After applying the inclusion and exclusion criteria, 577 patients were ultimately included for analysis. The inclusion criteria were as follows: (1) diagnosis of AML, acute lymphoblastic leukaemia (ALL), myelodysplastic syndrome (MDS), or non-Hodgkin lymphoma (NHL); (2) undergoing haplo-PBSCT; The exclusion criteria were as follow: (1) lack of post-transplant immune reconstitution data; (2) death within 1 month post-transplant; or (3) loss to follow-up. All donors were from HLA-haploidentical relatives, defined as 5/10 to 7/10 matched alleles with the recipient [18]. The final follow-up was completed in October 2024. This study was approved by the local ethics committee and conducted in accordance with the Declaration of Helsinki. Written informed consent was obtained from all participants.

### Transplant procedures

Patients with AML and MDS received conditioning regimens including busulfan, fludarabine and cytarabine [19]. Patients diagnosed with ALL and NHL were conditioned using total body irradiation (TBI)- or busulfan -based regimens [19]. Myeloablative conditioning (MAC) regimens were prescribed to patient with age <55 years old or hematopoietic stem cell transplantation comorbidity index (HCT-CI) ≤ 2, while reduced-intensity conditioning (RIC) were used for patients with age ≥55 years and HCT-CI ≥ 3 [20]. All patients received peripheral blood stem cell grafts mobilised with granulocyte colony-stimulating factor. Graft-versus-host disease (GVHD) prophylaxis included three regimens: anti-thymocyte globulin (ATG; 10 mg/kg) –based [21], low-dose ATG (5 mg/kg) plus low-dose posttransplant cyclophosphamide (PT-Cy; 50 mg/kg on day +3)-based [22] and PT-Cy –based [21].

### Definition and evaluation

Risk classification was disease-specific and uniformly categorised into three levels—low, intermediate and high—for statistical analysis. For AML, risk was defined as favourable (low), intermediate, or adverse (high) according to the 2022 European LeukemiaNet guidelines [23]. For MDS, stratification was based on the Revised International Prognostic Scoring System (IPSS-R): low risk (0–3), intermediate risk (>3–4.5) and high risk (>4.5) [24]. For ALL, risk was categorised into favourable (low risk) and adverse (high risk) groups according to the current National Comprehensive Cancer Network guidelines and institutional consensus [25]. For NHL, risk was assessed using a modified International Prognostic Index (IPI): low risk (score 0–1), intermediate risk (score 2–3) and high risk (score 4–5) [26].

Acute GVHD (aGVHD) and chronic GVHD (cGVHD) were graded according to the modified Glucksberg criteria for aGVHD and the 2014 NIH consensus criteria for cGVHD [27, 28]. TRM was defined as death due to causes unrelated to relapse, relapse rate (RR) was calculated as the cumulative incidence of relapse from the time of transplantation to the last follow-up, and OS was calculated as the proportion of patients who remained alive from the time of transplantation to the last follow-up, with death from any cause considered an event. For AML/ALL, relapse was defined as bone marrow blasts ≥5%, reappearance of leukaemic blasts in peripheral blood, or any extramedullary leukaemic infiltration. For MDS, relapse was defined as reappearance or persistence of disease with bone marrow blasts ≥5% (or progression to AML with blasts ≥20%), and/or reappearance of a patient-specific cytogenetic/molecular abnormality present before transplantation, confirmed on serial assessments where applicable. For NHL, relapse or progression was defined according to conventional lymphoma response criteria: biopsy-proven recurrent disease or imaging evidence of new or enlarging nodal/extranodal lesions on CT or PET-CT; for fluorodeoxyglucose (FDG)-avid subtypes, a Deauville score ≥4 or unequivocal radiologic progression was considered relapse/progression. Quantitative real-time PCR assays for cytomegalovirus (CMV) DNA and Epstein-Barr virus (EBV) DNA were conducted once or twice weekly in peripheral blood. Reactivation was defined as two consecutive positive PCR results with DNA copy numbers exceeding 1 × 10^3^ copies/mL, or a single result exceeding 5 × 10^3^ copies/mL [18].

### Flow cytometry

Peripheral blood samples from recipients were collected at 1, 2, 3, 4, 6, 9 and 12 months post-transplantation and analysed using multiparametric 8-colour flow cytometry with the following monoclonal antibody and fluorescent dyes: Panel 1: FITC-CD3, PE-CD16/56, ECD-CD19, PerCP-Cy5.5-CD45RA, APC-CD8, BV421-CD45RO, APC-Cy7-CD4, 7-AAD; Panel 2: FITC-CD3, PE-CD69, ECD-CD25, APC-Cy7-CD127, APC-HLA-DR, PerCP-Cy5.5-CD4, BV421-CD8, 7 -AAD (BD Biosciences, San Jose, CA and Beckman Coulter, Brea, CA). Lymphocyte subsets were defined as follows: Tregs, CD3⁺CD4⁺CD25⁺CD127^–^; naïve CD4⁺ T cells, CD3⁺CD4⁺CD45RA⁺; memory CD4⁺ T cells, CD3⁺CD4⁺CD45RO⁺; naïve CD8⁺ T cells, CD3⁺CD8⁺CD45RA⁺; memory CD8⁺ T cells, CD3⁺CD8⁺CD45RO⁺; early-activated T cells, CD3⁺CD69⁺; late-activated T cells, CD3⁺HLA-DR⁺; NK cells, CD3⁻CD16⁺CD56⁺; B cells, CD19⁺.

### Statistical analysis

All statistical analyses were performed using R software version 4.2.2 (R Foundation for Statistical Computing, Vienna, Austria). Baseline characteristics were summarised using descriptive statistics. Categorical variables were compared using Fisher’s exact test or chi-square test, while continuous variables were compared using the Wilcoxon rank-sum test. The primary endpoints of this study were OS, TRM and RR; secondary endpoints included aGVHD, cGVHD, CMV and EBV reactivation 100 days post-transplant. For each lymphocyte subset, absolute counts (AC) at 1, 2, 3, 4, 6, 9 and 12 months post-transplantation were dichotomised into high and low groups based on the median value. As a sensitivity analysis, each subset was also modelled as a continuous predictor at each time point. Univariate survival analysis was conducted to evaluate the association between lymphocyte reconstitution at each time point and transplant outcomes, including TRM, RR and OS. Additionally, the association of lymphocyte subset recovery with early CMV and EBV reactivation (within 100 days post-transplant), as well as the incidence of aGVHD and moderate-to-severe cGVHD, was also assessed.

Covariates included in the survival analyses were patient age, patient sex, donor age, donor sex, patient–donor sex, aGVHD occurrence, GVHD prophylaxis regimen, conditioning regimen, disease type, risk stratification, disease status at transplantation, CD34⁺ cell dose, CD3⁺ cell dose, Eastern Cooperative Oncology Group (ECOG) performance status, HCT-CI and CMV and EBV reactivation status. For analyses of early CMV reactivation, we additionally evaluated the effect of letermovir prophylaxis as a candidate covariate, and for each endpoint (OS, TRM, RR and moderate-to-severe cGVHD), systemic corticosteroid exposure (yes/no) before the occurrence of that endpoint was recorded (mostly initiated for treatment of aGVHD) and evaluated as a clinical covariate. Variables with a *P* < 0.05 in univariate analysis were included in the multivariate Cox proportional hazards regression model using a forward stepwise approach. The results of the multivariate analysis were considered primary for interpretation.

Causal mediation analysis based on the counterfactual framework was employed to evaluate whether early-phase CMV reactivation mediated the relationship between Treg reconstitution and TRM [29]. This approach decomposed the total effect of cell reconstitution (exposure) on TRM (outcome) into a direct effect and an indirect effect operating through a mediator—CMV reactivation. The direct and indirect effects, along with their 95% confidence intervals (CI), were estimated using quasi-Bayesian approximation methods implemented via the R package ‘mediation’. To further illustrate the relationships among immune cell reconstitution, CMV reactivation and TRM, a Sankey diagram was constructed for visual representation [30].

## Results

### Patient Characteristics

Five hundred and seventy-seven patients were enroled into the analysis, excluding 85 patients including 56 lacking post-transplant immune reconstitution data, 27 death within 1 month post-transplant and 2 without follow-up. AML accounted for 298 cases (51.6%), ALL for 104 (18.0%), MDS for 102 (17.7%) and NHL for 73 (12.7%). The median ages of patients and donors were 40 years (range: 6–71) and 32 years (range: 8–69), respectively. A total of 170 patients received ATG-based regimen, 340 received low-dose ATG plus low-dose PT-Cy-based regimen, and the remaining 67 were treated with PT-Cy-based regimen for GVHD prophylaxis. At transplantation, 445 patients were in complete remission, while 132 patients presented with active disease. The baseline characteristics of the cohort are summarised in Table 1.

**Table 1:** Patients’ characteristics.

Characteristic	All patients (*n* = 577)
Median patient age, year (range)	40 (6–71)
Median donor age, year (range)	32 (8–69)
Patient sex	
Male	346 (60.0%)
Female	231 (40.0%)
Donor sex	
Male	386 (66.9%)
Female	191 (33.1%)
Patient–donor sex	
Female to male	104 (18.0%)
Others	473 (82.0%)
GVHD prophylaxis	
ATG + CSA + MTX + MPA	170 (29.5%)
ATG + CSA + CTX + MPA	340 (58.9%)
CTX + CSA + MPA	67 (11.6%)
Conditioning regimens	
MAC	439 (76.1%)
RIC	138 (23.9%)
Diagnosis	
AML	298 (51.6%)
ALL	104 (18.0%)
MDS	102 (17.7%)
NHL	73 (12.7%)
Risk stratification	
Low risk	107 (18.5%)
Intermediate risk	152 (26.3%)
High risk	318 (55.1%)
Disease status	
CR	445 (77.1%)
NR	132 (22.9%)
ECOG	
0	392 (67.9%)
1	162 (28.1%)
2	15 (2.6%)
3–4	8 (1.4%)
HCT-CI	
<3	550 (95.3%)
≥3	27 (4.7%)
Median CD34+ cell (×10^6^/kg), range	11.12 (1.58–46.55)
Median CD3 + T cell (×10^8^/kg), range	3.30 (0.61–10.8)

*GvHD* graft-versus-host disease, *CSA* cyclosporine, *MTX* methotrexate, *MPA* mycophenolate, *ATG *rabbit-anti-human thymocyte globulin, *CTX* cyclophosphamide, *MAC* myeloablative conditioning, *RIC* reduced-intensity conditioning, *AML* acute myeloid leukaemia, *ALL* acute lymphoblastic leukaemia, *MDS* myelodysplasia syndrome, *NHL* non-Hodgkin lymphoma, *CR* complete remission, *NR* non-complete remission, *ECOG* Eastern cooperative oncology group, *HCT-CI* hematopoietic cell transplantation comorbidity index.

### Transplant outcomes

All enroled patients achieved successful engraftment, and those with active disease at transplantation achieved complete remission post-transplant. After a median follow-up of 24 months (range: 1–88 months), the 5-year OS was 72.0% (95% CI: 67.3–77%, *n* = 137). The 5-year of TRM was 14.7% (95% CI:10.1–19.0%, *n* = 64), while the 5-year cumulative incidence of relapse 21.7% (95% CI: 17.8–25.4%, *n* = 108). The cumulative incidence of grade I–IV aGVHD was 30.9% (95% CI:27.0–34.6%, *n* = 178), while grade II–IV aGVHD occurred in 14.7% of patients (95% CI:11.6–17.7%,*n* = 76). The 2-year cumulative incidence of moderate-to-severe cGVHD was 21.7% (95% CI: 18.0–25.1%, *n* = 114). Within 100 days post-transplantation, the cumulative incidence of CMV reactivation was 34.3% (95% CI:30.3–38.2%, *n* = 192), and EBV reactivation was 38.8% (95% CI: 34.6–42.7%, *n* = 216).

### Lymphocyte subset reconstitution

**Fig. 1:**
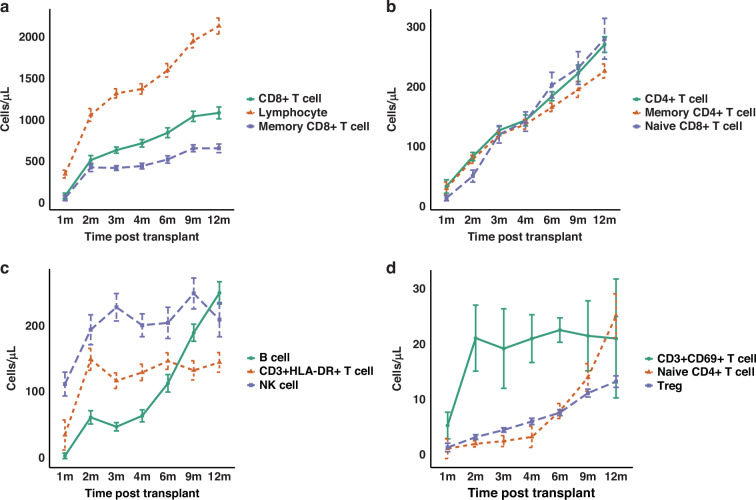
Reconstitution kinetics of lymphocyte subsets following haploidentical PBSCT. Median absolute counts (cells/μL) of each lymphocyte subset were plotted over time from 1 to 12 months post-transplantation. **a** Total lymphocytes, CD8⁺ T cells and memory CD8⁺ T cells. **b** CD4⁺ T cells, memory CD4⁺ T cells, and naïve CD8⁺ T cells. **c** CD3⁺HLA-DR⁺ late-activated T cells, NK cells, and B cells. **d** CD3⁺CD69⁺ early-activated T cells, naïve CD4⁺ T cells, and regulatory T cells (Tregs).

Distinct reconstitution patterns were observed across lymphocyte subsets post-transplantation. Total lymphocytes showed the fastest and strongest recovery, surpassing 2000 cells/μL at 12 months, whereas CD8^+^ T cells and memory CD8^+^ T cells exhibited a more gradual recovery trajectory (Fig. 1a). CD4^+^ T cells, memory CD4^+^ T cells and naïve CD8^+^ T cells demonstrated synchronised, linear increases, with CD4^+^ T cells and naïve CD8^+^ T cells reaching comparable levels (~270 cells/μL) at 12 months, although memory CD4^+^ T cells expanded more slowly in the later phase (Fig. 1b). CD3^+^HLA-DR^+^ late-activated T cells showed rapid and sustained reconstitution, while NK cells peaked early and fluctuated moderately thereafter. B cells displayed delayed but robust reconstitution, indicative of prolonged maturation processes (Fig. 1c). Early-activated CD3^+^CD69^+^ T cells expanded rapidly shortly after transplantation, whereas naïve CD4^+^ T cells increased sharply after 6 months, suggesting thymic regeneration. Tregs showed gradual, steady growth throughout the follow-up period (Fig. 1d). Additionally, a marked inversion of the CD4/CD8 T cell ratio was observed in the early post-transplant period, reaching its lowest median value at month 2 (0.16), followed by a gradual recovery over time. Specifically, the median CD4/CD8 ratio was 0.40, 0.16, 0.20, 0.21, 0.21, 0.23 and 0.25 at months 1, 2, 3, 4, 6, 9 and 12, respectively.

### Impact of GVHD prophylaxis on immune reconstitution and outcomes

We further compared lymphocyte reconstitution across the three GVHD prophylaxis regimens (ATG + PT-Cy, ATG alone and PT-Cy alone). As shown in Fig. S1, prophylaxis significantly affected the recovery of several subsets at multiple time points. PT-Cy alone was associated with higher counts of naïve CD4⁺ T cells, naïve CD8⁺ T cells and Tregs from the early to late phases (1–12 months), whereas ATG alone showed higher early NK-cell counts (particularly at 1–2 months). Importantly, despite these effects on reconstitution, the prophylaxis regimens themselves did not influence OS, TRM, or RR in this cohort (all *p* > 0.05).

### CD4^+^ T cell reconstitution and survival outcomes

**Fig. 2:**
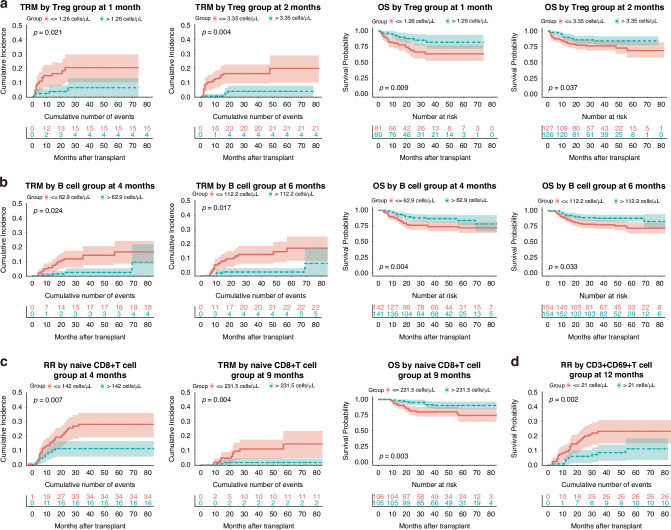
Key associations between lymphocyte subset reconstitution and transplant outcomes. **a** Tregs: cumulative incidence of TRM at 1 and 2 months, and OS at 1 and 2 months, stratified by median Treg counts. **b** B cells: cumulative incidence of TRM at 4 and 6 months, and OS at 4 and 6 months, stratified by median B-cell counts. **c** Naïve CD8⁺ T cells: cumulative incidence of RR at 4 months, TRM at 9 months and OS at 9 months, stratified by median naïve CD8⁺ T-cell counts. **d** CD3⁺CD69⁺ T cells: cumulative incidence of RR at 12 months, stratified by median CD3⁺CD69⁺ T-cell counts. At each indicated month, subsets were dichotomised by the median absolute count (cells/μL). *P* values shown in each panel are from multivariable Cox proportional hazards models. Curves are displayed as Kaplan–Meier (OS) or cumulative incidence (TRM/RR) for visualisation; OS panels include numbers at risk, and TRM/RR panels include cumulative numbers of events. Shaded areas represent 95% confidence intervals, and tick marks indicate censoring. OS overall survival, TRM transplant-related mortality, RR relapse rate.

Superior CD4^+^ T cell recovery at 4 months correlated with improved OS (Fig. S2A, Table 2), and better reconstitution at 9 months was associated with reduced RR (Fig. S2C, Table 4). Among CD4^+^ T cell subsets, enhanced naïve CD4^+^ T cell counts at 1 month reduced RR (Fig. S2C, Table 4). Higher memory CD4^+^ T cell counts at 4 months improved OS (Fig. S2A, Table 2), while higher AC at 9 and 12 months correlated with decreased relapse risk (Fig. S2C, Table 4). Furthermore, superior reconstitution of Tregs at 1, 2 and 4 months significantly reduced TRM, thereby improving OS (Fig. 2a, S2A, B, Tables 2, 3).

**Table 2:** Association between lymphocyte subset reconstitution and overall survival.

Lymphocyte subsets	Median (cells/μL)	5-year OS (95% CI)		HR (95% CI)	*P*
		>median	<median		
CD4^+^ T cell at 4 m	144	78.8% (68.6–90.7%)	71.7% (63.4–81.1%)	0.53 (0.31–0.89)	0.021
memory CD4^+^ T cell at 4 m	137.4	78.4% (67.8–90.7%)	72.0% (63.8–81.3%)	0.54 (0.31–0.92)	0.024
Treg at 1 m	1.3	81.7% (71.7–93.2%)	63.5% (52.3–77.1%)	0.36 (0.17–0.75)	0.009
Treg at 2 m	3.4	84.1% (77.4–91.3%)	68.8% (57.8–81.9%)	0.54 (0.30–0.97)	0.037
Treg at 4 m	6	86.3% (80.0–93.1%)	73.0% (62.7–85.1%)	0.45 (0.23–0.87)	0.016
CD8^+^ T cell at 9 m	1033	90.6% (84.8–96.7%)	73.8% (63.9–85.3%)	0.39 (0.18–0.85)	0.019
naïve CD8^+^ T cell at 4 m	142	81.0% (71.9–91.3%)	69.2% (60.0–79.7%)	0.47 (0.27–0.80)	0.008
naïve CD8^+^ T cell at 9 m	231.5	90.3% (84.4–96.6%)	74.3% (64.4–85.8%)	0.30 (0.14–0.66)	0.003
NK cell at 9 m	246.2	91.3% (86.0–96.9%)	72.7% (62.5–84.7%)	0.43 (0.20–0.95)	0.035
B cell at 4 m	62.9	78.0% (66.0–92.1%)	71.7% (63.7–80.5%)	0.43 (0.24–0.76)	0.004
B cell at 6 m	112.2	82.6% (72.3–94.4%)	71.7% (63.3–81.1%)	0.53 (0.30–0.95)	0.033

**Table 3:** Association between lymphocyte subset reconstitution and transplant-related mortality.

Lymphocyte subsets	Median (cells/μL)	5-year TRM (95% CI)		HR (95% CI)	*P*
		> median	<median		
Treg at 1 m	1.3	6.7% (0–13.2%)	20.5% (10.3–29.6%)	0.20 (0.09–0.82)	0.021
Treg at 2 m	3.4	4.1% (0.04–8.0%)	19.9% (10.0–28.8%)	0.20 (0.07–0.58)	0.004
Treg at 4 m	6	3.0% (0–6.2%)	12.0% (5.1–18.3%)	0.24 (0.07–0.81)	0.028
naïve CD8^+^ T cell at 1 m	14.1	15.5% (2.0–27.1%)	20.2% (10.0–29.4%)	0.34 (0.13–0.87)	0.025
naïve CD8^+^ T cell at 3 m	120.6	10.5% (1.3–18.8%)	14.6% (8.5–20.3%)	0.40 (0.18–0.87)	0.020
naïve CD8^+^ T cell at 9 m	231.5	1.9% (0–4.5%)	14.7% (4.8–23.5%)	0.11 (0.02–0.50)	0.004
NK cell at 2 m	191.5	5.7% (1.8–9.4%)	17.5% (9.9–24.5%)	0.35 (0.16–0.80)	0.012
B cell at 2 m	60.2	6.5% (2.3–10.5%)	16.3% (9.2–22.7%)	0.40 (0.18–0.89)	0.025
B cell at 3 m	46.2	4.8% (1.2–8.3%)	22.0% (11.4–31.4%)	0.38 (0.14–0.99)	0.048
B cell at 4 m	62.9	9.5% (0–21.9%)	16.7% (8.5–24.2%)	0.28 (0.10–0.80)	0.024
B cell at 6 m	112.2	8.4% (0–18.9%)	18.5% (10.0–26.1%)	0.30 (0.11–0.80)	0.017
B cell at 9 m	186	5.3% (0–13.4%)	11.5% (4.8–17.7%)	0.21 (0.05–0.90)	0.044

### CD8^+^ T Cell Reconstitution and Survival Outcomes

Higher CD8^+^ T cell AC at 9 months post-transplantation was associated with better OS (Fig. S2A, Table 2), and higher counts at 9 and 12 months correlated with lower relapse risk (Fig. S2C, Table 4). For CD8^+^ T cell subsets, superior reconstitution of naïve CD8^+^ T cells at 1, 3 and 9 months significantly reduced TRM (Fig. 2c and Fig. S2B, Table 3), and better reconstitution at 4 and 9 months improved OS (Fig. 2c and Fig. S2A, Table 2). Moreover, higher naïve CD8^+^ T cell AC at 4 months was significantly associated with reduced RR (Fig. 2c, Table 4).

**Table 4:** Association between lymphocyte subset reconstitution and relapse rate.

Lymphocyte subsets	Median (cells/μL)	5-year RR (95% CI)		HR (95% CI)	*P*
		>median	<median		
CD4^+^ T cell at 9 m	222.5	11.7% (4.9–18%)	22.9% (14.1–30.7%)	0.44 (0.21–0.94)	0.029
naive CD4 + T cell at 1 m	21	14.2% (5.2–22.2%)	30.2% (17.2–41.2%)	0.35 (0.16–0.79)	0.011
memory CD4^+^ T cell at 9 m	195.4	9.6% (3.4–15.5%)	25.3% (16.1–33.5%)	0.34 (0.17–0.74)	0.006
memory CD4^+^ T cell at 12 m	226.4	11.3% (5.0–17.2%)	23.8% (14–32.4%)	0.48 (0.24–0.96)	0.039
CD8^+^ T cell at 9 m	1033	11.1% (4.6–17.2%)	23.8% (14.7–32%)	0.48 (0.23–0.99)	0.049
CD8^+^ T cell at 12 m	1079.9	12.0% (4.6–18.7%)	22.0% (13.9–29.3%)	0.40 (0.20–0.81)	0.013
naïve CD8^+^ T cell at 4 m	142	11.5% (6.0–16.7%)	28.2% (19.3–36.2%)	0.43 (0.23–0.79)	0.007
CD3^+^ CD69^+^ T cell at 9 m	21.4	7.7% (2.4–12.8%)	26.3% (16.9–34.6%)	0.31 (0.15–0.64)	0.004
CD3^+^ CD69^+^ T cell at 12 m	21	11.2% (3.6–18.3%)	23.2% (14.9–30.8%)	0.34 (0.17–0.69)	0.002

### B cell reconstitution and survival outcomes

Enhanced B cell reconstitution at 2, 3, 4, 6 and 9 months post-transplantation was associated with significantly lower TRM (Fig. 2b and Fig. S2B, Table 3). Additionally, higher B cell counts at 4 and 6 months correlated with improved OS (Fig. 2b, Table 2).

### Other subsets reconstitution and survival outcomes

**Fig. 3:**
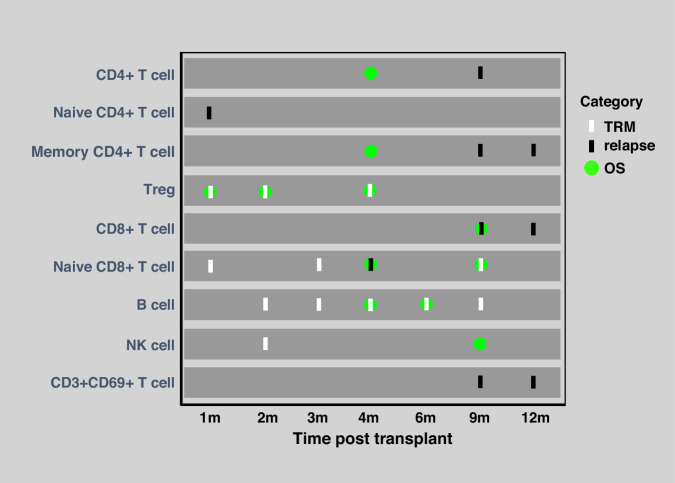
Summary of lymphocyte subsets significantly associated with transplant outcomes at each time point post-transplantation. Each coloured symbol represents a statistically significant association between a given lymphocyte subset and clinical outcome. White bars indicate reduced TRM, black bars indicate reduced relapse risk and green circles indicate improved OS. Outcomes are mapped across time points (1–12 months) for each lymphocyte subset. TRM transplant-related mortality, OS overall survival.

Better NK cell reconstitution at 2 months was associated with reduced TRM (Fig. S2B, Table 3), and superior NK cell counts at 9 months improved OS (Fig. S2A, Table 2). Additionally, higher CD3^+^CD69^+^ T cell counts at 9 and 12 months correlated significantly with lower RR (Fig. 2d and Fig. S2C, Table 4). Figure 3 summarises the lymphocyte subsets significantly associated with transplant outcomes at each post-transplantation time point. For completeness, the full results of the univariate and multivariate analyses for all lymphocyte subsets at each time point, including non-significant findings, are provided in Tables S1–S18. Moreover, in sensitivity analyses treating subsets as continuous variables, the overall patterns were highly consistent with the median-based findings (Tables S19–S21).

### Influence of lymphocyte subsets on other transplant outcomes

**Fig. 4:**
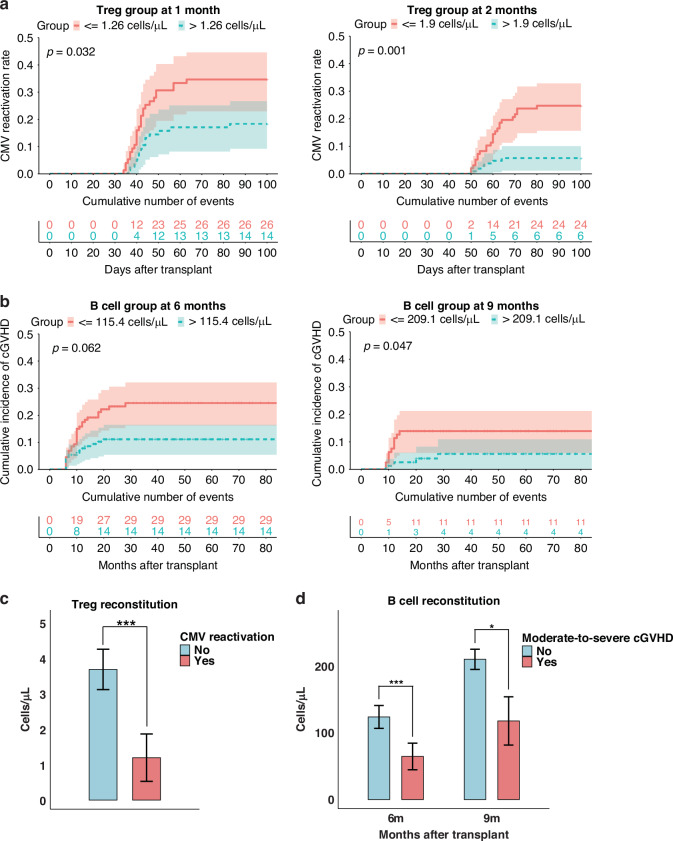
Influence of lymphocyte subset reconstitution on CMV reactivation and moderate-to-severe cGVHD. **a** Cumulative incidence of CMV reactivation stratified by Treg reconstitution at 1 and 2 months (dichotomised by the median absolute count, cells/μL). *P* values are from multivariable cause-specific Cox models adjusted as specified in Methods (including letermovir prophylaxis). **b** Cumulative incidence of moderate-to-severe cGVHD according to B-cell reconstitution at 6 and 9 months (median split). *P* values are from multivariable cause-specific Cox models that include systemic steroid use and other prespecified covariates (Methods). **c** Comparison of Treg absolute counts measured prior to CMV reactivation between patients with and without early CMV reactivation (bars show mean ± SEM). *P* values are from Wilcoxon rank-sum tests. **d** Comparison of B-cell absolute counts at 6 and 9 months (measured prior to clinical cGVHD) between patients with and without subsequent moderate-to-severe cGVHD (mean ± SEM). *P* values are from Wilcoxon rank-sum tests. Asterisks denote statistical significance: *P* < 0.05 (*), *P* < 0.01 (**), *P* < 0.001 (***). CMV cytomegalovirus, cGVHD chronic graft-versus-host disease, SEM standard error of the mean.

We further investigated whether lymphocyte reconstitution influenced post-transplant complications that may contribute to TRM, including viral reactivations and GVHD. Cox analyses showed that superior Treg reconstitution at 1 and 2 months was associated with a lower risk of early CMV reactivation. At 1 month, CMV reactivation within 100 days occurred in 19.7% (95% CI, 10.3–28.2%) of patients with Treg > median versus 34.2% (22.6–44.1%) with Treg ≤ median; letermovir prophylaxis itself was strongly protective (HR 0.20, 95% CI 0.09–0.45; *p* < 0.001), and higher Treg counts remained independently protective after adjustment (HR 0.49, 95% CI 0.26–0.94; *p* = 0.032; Fig. 4a, Table S22). At 2 months, the corresponding incidences were 5.7% (1.2–10.1%) vs 24.7% (15.6–32.9%), and Treg reconstitution continued to show an independent association (HR 0.22, 95% CI 0.09–0.55; *p* = 0.001; Fig. 4a), while letermovir also remained significant (HR 0.37, 95% CI 0.16–0.87; *p* = 0.022, Table S23). In univariate analyses, NK reconstitution at 2 months was associated with a lower risk of CMV reactivation; the 100-day cumulative incidence was 9.6% (95% CI, 4.0–14.8%) in NK > median versus 19.5% (11.8–26.4%) in NK ≤ median (HR 0.47, 95% CI 0.23–0.96; *p* = 0.039). However, after adjustment for letermovir and other covariates, this association was attenuated and became non-significant (HR 0.59, 95% CI 0.23–1.53; *p* = 0.279, Table S23). In contrast, we did not find any association between early B cell or naïve CD8^+^ T cell reconstitution and CMV reactivation, nor did we observe any correlation between lymphocyte subsets reconstitution and EBV reactivation. Furthermore, early reconstitution of the above-mentioned lymphocyte subsets showed no significant correlation with the incidence of grades I–IV or II–IV aGVHD.

In univariate analyses, higher B-cell counts were associated with lower subsequent cGVHD at 2 months (HR 0.50, 95% CI 0.27–0.94; *p* = 0.031), 6 months (HR 0.48, 95% CI 0.26–0.90; *p* = 0.022) and 9 months (HR 0.27, 95% CI 0.09–0.81; *p* = 0.019). The group-wise cumulative incidences for B cells> vs ≤median were 11.2% (95% CI 5.6–16.4) vs 19.5% (12.6–25.9) at 2 months, 11.8% (6.0–17.2) vs 22.9% (15.0–30.1) at 6 months and 5.5% (0–10.7) vs 17.3% (8.6–25.2) at 9 months. In multivariable models including systemic steroid use, the association at 2 months was not significant (B cells> vs ≤median: HR 1.08, 95% CI 0.55–2.13; *p* = 0.828; systemic steroid use: HR 5.64, 95% CI 2.46–12.9; *p* < 0.001, Table S24). At 6 months, higher B cells showed a borderline association with lower cGVHD (HR 0.54, 95% CI 0.28–1.03; *p* = 0.062, Fig. 4b), whereas systemic steroid use remained associated with increased risk (HR 6.17, 95% CI 2.58–14.8; *p* < 0.001, Table S25). At 9 months, higher B cells were independently associated with lower cGVHD (HR 0.32, 95% CI 0.10–0.99; *p* = 0.047; Fig. 4b and Table S26). Additionally, patients without CMV reactivation had significantly higher AC of Tregs (3.7 cells/μL *vs*. 1.2 cells/μL, *p* < 0.001, Fig. 4c) compared with those who developed CMV reactivation, measured prior to reactivation. Similarly, patients who did not develop cGVHD demonstrated significantly higher B cell counts at 6 months (124.5 cells/μL *vs*. 65.0 cells/μL, *p* < 0.001, Fig. 4d) and 9 months (211.4 cells/μL *vs*. 118.4 cells/μL, *p* = 0.011, Fig. 4d) compared to those who developed cGVHD, measured prior to cGVHD. However, we found no significant association between Treg, NK cell, or naïve CD8+ T cell reconstitution and moderate-to-severe cGVHD.

### Mediation analysis

**Fig. 5:**
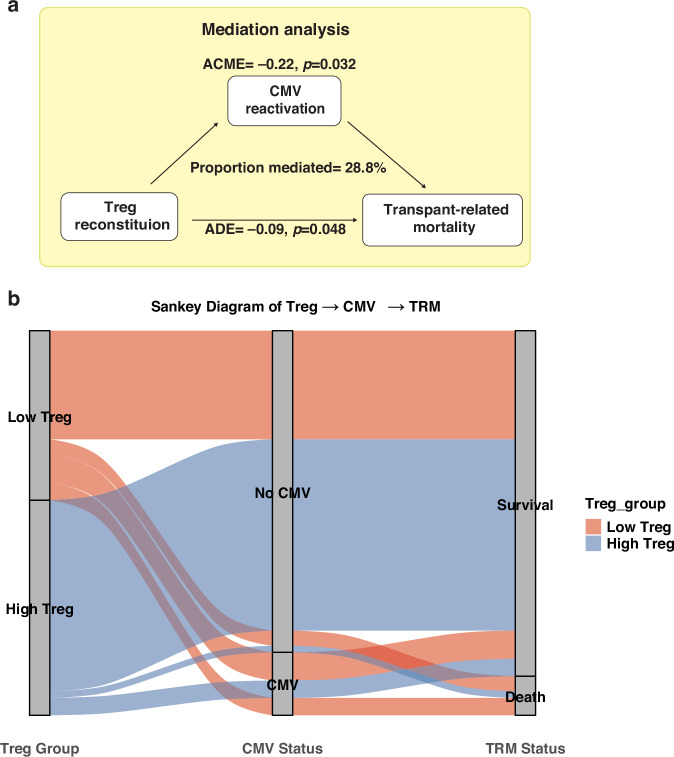
Mediation effect of early CMV reactivation on the relationship between Treg reconstitution and TRM. **a** Mediation analysis showing that early CMV reactivation significantly mediated the association between Treg reconstitution at 2 months and TRM (ACME = –0.22, *p* = 0.032), accounting for 28.8% of the total effect. A significant direct effect of Treg reconstitution on TRM was also observed (ADE = –0.09, *p* = 0.048). **b** Sankey diagram illustrating the distribution of patients across Treg reconstitution groups (low vs. high), CMV reactivation status (no CMV vs. CMV), and transplant-related mortality (survival vs. death). Patients with high Treg reconstitution were more likely to avoid CMV reactivation and had a lower incidence of TRM. TRM transplant-related mortality, ACME average causal mediation effect, ADE average direct effect.

In the Cox proportional hazards regression analysis evaluating the association between immune reconstitution status at 2 months post-transplant and TRM, early CMV reactivation emerged as an independent factor associated with TRM (9.7% in patients without CMV reactivation *vs*. 18.1% in those with reactivation; *p* = 0.038 when analysed with Treg reconstitution, Table S8). These findings led us to hypothesise that improved early Treg reconstitution may reduce TRM by suppressing early CMV reactivation. To test this hypothesis, we performed a mediation analysis to estimate the direct and indirect effects. A causal pathway among Treg reconstitution, CMV reactivation and TRM was identified (Fig. 5a, b), in which early CMV reactivation mediated the effect of Treg reconstitution at 2 months on TRM (ACME = –0.22, *p* = 0.032), accounting for 28.8% of the total effect. In addition, a significant direct effect of Treg reconstitution on TRM was confirmed (ADE = –0.09, *p* = 0.048). Furthermore, we evaluated the potential causal relationship between B cell reconstitution (at 6 and 9 months) and TRM via cGVHD, but found no evidence supporting a mediating role of cGVHD in this context.

## Discussion

Our study identified significant associations between lymphocyte subset reconstitution and clinical outcomes following haplo-PBSCT. Overall CD4⁺ T cell recovery at 9 months post-transplantation was associated with reduced relapse risk, primarily driven by memory CD4⁺ T cells. Among CD4⁺ subsets, Tregs exerted the most pronounced protective effect; higher Treg counts at 1, 2 and 4 months post-transplant significantly reduced TRM, thereby improving OS, without increasing the risk of relapse. Enhanced reconstitution of CD8⁺ T cells, especially naïve subsets, was also associated with lower TRM, decreased RR and improved OS. Notably, B cell reconstitution post-transplantation consistently correlated with reduced TRM, contributing to improved OS. In addition, elevated CD3⁺CD69⁺ early-activated T cell levels at 9 and 12 months post-transplant were significantly associated with reduced relapse incidence. Furthermore, higher Treg counts at 1 and 2 months post-transplant were significantly associated with a lower incidence of early CMV reactivation, while B cell reconstitution at 6 and 9 months post-transplant markedly reduced the occurrence of moderate-to-severe cGVHD. Mediation analysis confirmed that improved Treg reconstitution at 2 months post-transplant reduced TRM, in part by suppressing early CMV reactivation.

The lymphocyte subset reconstitution patterns in our haploidentical HSCT cohort align with established trends in post-transplant immune recovery [4, 5, 31]. NK cells reconstituted rapidly, reaching normal levels within the first month, consistent with their innate immune role. In contrast, adaptive immunity recovered more gradually. CD8⁺ T cells rebounded faster than CD4⁺ T cells, causing a sustained CD4/CD8 ratio inversion, which reached a nadir at month 2 and gradually normalised by month 12. B cell reconstitution was delayed but robust, with a notable rise starting around month 6 and normalising by month 9, reflecting their dependency on prolonged maturation and follicular helper T cell support [31].

Although immune reconstitution has been widely studied in relation to survival outcomes, comprehensive analyses in haplo-PBSCT remain limited. We revealed that higher CD4⁺ T cell reconstitution at 4 months post-transplant was linked to improved OS, while recovery at 9 months reduced RR, primarily through memory CD4⁺ T cells. In contrast, higher naïve CD4⁺ T cell counts at 1 month post-transplant were associated with lower relapse risk. While some studies have linked early CD4⁺ T cell recovery to reduced relapse, others, including Kim et al. and Bejanyan et al., reported associations with improved OS or TRM but not relapse [32, 33]. Our results help bridge this inconsistency by highlighting Tregs as a CD4⁺ subset with a clear protective role—reducing TRM at 1, 2 and 4 months post-transplant without affecting relapse. Most available work in allogeneic HSCT has focused on the immunoregulatory role of Tregs in limiting GVHD and maintaining immune homoeostasis, rather than on survival endpoints [14–16]. We found that Treg reconstitution at 1 and 2 months post-transplant significantly reduced early phase CMV reactivation. The link between Tregs and CMV control remains unclear. Sarvari et al. reported no association between Treg reconstitution and CMV reactivation [34], and Domenico et al. highlighted a connection between Treg recovery and CMV-specific CD8⁺ T cell restoration [35]. A murine study also showed that Tregs suppressed CMV reactivation in tissue-specific patterns [36]. In our cohort, infectious pneumonia—particularly CMV pneumonitis—was a leading cause of TRM, supporting the hypothesis that Tregs may reduce TRM by limiting CMV reactivation. Mediation analysis confirmed that Treg reconstitution at 2 months post-transplant significantly reduced TRM, partially through decreasing early CMV reactivation. This analysis established a mechanistic link between early Treg recovery, CMV control and reduced TRM, emphasising the critical role of early Tregs in preventing opportunistic infections and enhancing post-transplant survival.

Better CD8⁺ T cell reconstitution at 9 and 12 months post-transplant was associated with reduced relapse, while early and sustained reconstitution of naïve CD8⁺ T cells correlated with improved OS, lower TRM and reduced relapse. CD8⁺ T cells are central to anti-viral and anti-leukaemic immunity, yet studies on their post-transplant recovery have been inconsistent. In haploidentical settings, Tian et al. found that higher CD8⁺ T cell counts were linked to lower TRM and longer leukaemia-free survival but not relapse [37]. Ranti et al. and Yakoub-Agha et al. emphasised the importance of CD8⁺ T cell subsets, such as CD28⁻ cells, in predicting relapse risk [11, 38], but these findings warrant further investigation in haplo-PBSCT.

Robust B cell reconstitution was strongly associated with reduced TRM and improved OS. Previous studies have linked adequate B-cell recovery with lower infection risk and better survival [17, 39, 40], and some have reported higher relapse and mortality with low B-cell counts [13, 17], although others did not find a direct link with relapse [40]. While prior work has shown that cGVHD can delay B-cell reconstitution [41], whether B-cell recovery itself influences cGVHD incidence has been unclear. In our cohort, better B-cell reconstitution was associated with a lower incidence of moderate-to-severe cGVHD. After adjusting for systemic corticosteroid exposure, this protective association was absent at 2 months, borderline at 6 months and re-emerged at 9 months. Systemic steroid exposure itself was strongly associated with increased cGVHD risk in our data, consistent with evidence that preceding aGVHD—a common indication for steroid use—is a major risk factor for cGVHD [42–44]. Glucocorticoids also impair B-cell number and function, offering a plausible mechanism that early steroid exposure attenuates B-cell recovery signals until later stages [45]. Mediation analysis revealed no evidence that cGVHD mediated the relationship between B-cell reconstitution and TRM, suggesting independent protective mechanisms.

Better NK cell reconstitution at 2 months post-transplant was associated with lower TRM, aligning with prior studies [9, 10, 46]. However, despite the reported anti-leukaemic potential of NK cells [40, 47], our data showed no significant link between NK cell counts and relapse, which may reflect the use of PT-Cy–based GVHD prophylaxis in most patients. PT-Cy selectively eliminates mature donor NK cells and favours the emergence of immature subsets, blunting NK-mediated alloreactivity and graft-versus-leukaemia effects [48–50]. In contrast, higher CD3⁺CD69⁺ early-activated T cell levels at 9 and 12 months were associated with significantly reduced relapse. CD69 identifies T cells with recent activation, potentially contributing to anti-leukaemic responses. Although direct evidence on CD3⁺CD69⁺ T cells in HSCT is limited, our findings suggest they may serve as a potential biomarker for relapse risk.

Notably, although GVHD prophylaxis regimens significantly affected immune reconstitution dynamics, especially for Tregs, naïve CD4⁺/CD8⁺ T cells and NK cells, they did not affect OS, TRM, or RR in our cohort. This dissociation suggests that while certain regimens promote faster immune subset recovery, clinical outcomes may depend more on the quality and function of reconstituted cells rather than quantity alone. Moreover, the compensatory effects of supportive care, anti-infective prophylaxis and the intrinsic redundancy in immune recovery pathways may mitigate differences in outcomes. These findings align with recent perspectives that immunological fitness post-transplant is shaped by complex interactions beyond simple numeric thresholds [51].

In our study, some associations were significant at 1 month but not at nearby months. This may be due to several factors. First, each monthly landmark involves a different risk set, as patients with earlier events or missing samples are excluded, altering baseline risk and power. Second, ‘high’ and ‘low’ groups are defined by month-specific medians, so cut-offs and group membership shift over time. Third, events and time-dependent treatments such as systemic steroids are unevenly distributed, potentially confounding nearby months differently. Finally, biological and technical factors—including rapid NK peaks, slower B-cell maturation, thymic recovery of naïve T cells, assay variability and intermittent missingness—can further reduce consistency. For these reasons, single-month *P* values should be interpreted in the context of nearby patterns. In our cohort, the two most consistent trends were that higher Treg counts at 1–4 months and higher B-cell counts at 2–9 months both associated with lower TRM, supporting emphasis on biologically plausible and persistent signals.

In conclusion, our study provides a comprehensive characterisation of lymphocyte subset reconstitution following haplo-PBSCT and their associations with key transplant outcomes. Among all subsets, Treg reconstitution at 1 and 2 months emerged as a pivotal determinant of post-transplant prognosis. Through causal mediation analysis, we identified a mechanistic pathway in which improved Treg recovery significantly reduced TRM, in part by suppressing early CMV reactivation. This underscores the central role of early Treg reconstitution in infection control and enhancing OS. We also highlight the impact of B cell reconstitution in reducing both TRM and cGVHD, and the potential prognostic value of CD3⁺CD69⁺ T cells and naïve CD8⁺ T cells in relapse prevention. These results support dynamic immune monitoring and suggest early immune profiles may guide risk-adapted interventions in haploidentical transplantation.

## Supplementary information

Supplementary material

## Data Availability

The datasets analysed during the current study are available from the corresponding author on reasonable request.

## References

[1] Astigarraga CC, Mpms K, Iovino L, Milano F. Haploidentical transplantation: an optimal platform for graft manipulation and cellular therapies. Blood Rev. 2025;72:101286. 10.1016/j.blre.2025.101286 40133165

[2] Sugita J, Morita K, Konuma T, Yanada M. Allogeneic hematopoietic cell transplantation from alternative donors in acute myeloid leukemia. Ann Hematol. 2024;103:4851–68. 39153145 10.1007/s00277-024-05944-0

[3] Khan MA, Bashir Q, Chaudhry Q-u-N, Ahmed P, Satti TM, Mahmood SK. Review of haploidentical hematopoietic cell transplantation. J Glob Oncol. 2018;4:1–13. 30521413 10.1200/JGO.18.00130 PMC7010419

[4] Ogonek J, Juric MK, Ghimire S, Varanasi PR, Holler E, Greinix H, et al. Immune reconstitutional after allogeneic hematopoietic stem cell transplantation. Front Immunol. 2016;7:507. 27909435 10.3389/fimmu.2016.00507 PMC5112259

[5] Bosch M, Khan FM, Storek J. Immune reconstitution after hematopoietic cell transplantation. Curr Opin Hematol. 2012;19:324–35. 22517587 10.1097/MOH.0b013e328353bc7d

[6] Le Blanc K, Barrett AJ, Schaffer M, Hägglund H, Ljungman P, Ringdén O, et al. Lymphocyte recovery is a major determinant of outcome after matched unrelated myeloablative transplantation for myelogenous malignancies. Biol Blood Marrow Transplant. 2009;15:1108–15. 19660724 10.1016/j.bbmt.2009.05.015 PMC3793397

[7] Savani BN, Mielke S, Rezvani K, Montero A, Yong AS, Wish L, et al. Absolute lymphocyte count on day 30 is a surrogate for robust hematopoietic recovery and strongly predicts outcome after T cell-depleted allogeneic stem cell transplantation. Biol Blood Marrow Transplant. 2007;13:1216–23. 17889359 10.1016/j.bbmt.2007.07.005 PMC3426353

[8] Savani BN, Mielke S, Adams S, Uribe M, Rezvani K, Yong AS, et al. Rapid natural killer cell recovery determines outcome after T-cell-depleted HLA-identical stem cell transplantation in patients with myeloid leukemias but not with acute lymphoblastic leukemia. Leukemia. 2007;21:2145–52. 17673900 10.1038/sj.leu.2404892

[9] Buehlmann L, Buser AS, Cantoni N, Gerull S, Tichelli A, Gratwohl A, et al. Lymphocyte subset recovery and outcome after T-cell replete allogeneic hematopoietic SCT. Bone Marrow Transplant. 2011;46:1357–62. 21113185 10.1038/bmt.2010.306

[10] Minculescu L, Marquart HV, Friis LS, Petersen SL, Schiodt I, Ryder LP, et al. Early natural killer cell reconstitution predicts overall survival in T cell-replete allogeneic hematopoietic stem cell transplantation. Biol Blood Marrow Transplant. 2016;22:2187–93. 27664326 10.1016/j.bbmt.2016.09.006

[11] Ranti J, Kurki S, Salmenniemi U, Putkonen M, Salomäki S, Itälä-Remes M. Early CD8+-recovery independently predicts low probability of disease relapse but also associates with severe GVHD after allogeneic HSCT. PLoS ONE. 2018;13:13. 10.1371/journal.pone.0204136 PMC6147489 30235281

[12] van Roessel I, Prockop S, Klein E, Boulad F, Scaradavou A, Spitzer B, et al. Early CD4+T cell reconstitution as predictor of outcomes after allogeneic hematopoietic cell transplantation. Cytotherapy. 2020;22:503–10. 32622752 10.1016/j.jcyt.2020.05.005 PMC7484404

[13] Lucas AGT, Lindemans CA, Bhoopalan SV, Dandis R, Prockop SE, Naik S, et al. Early immune reconstitution as predictor for outcomes after allogeneic hematopoietic cell transplant; a tri-institutional analysis. Cytotherapy. 2023;25:977–85. 37330731 10.1016/j.jcyt.2023.05.012 PMC10984694

[14] Guo WW, Su XH, Wang MY, Han MZ, Feng XM, Jiang EL. Regulatory T cells in GVHD therapy. Front Immunol. 2021;12:697854. 10.3389/fimmu.2021.697854 PMC8250864 34220860

[15] Di Ianni M, Falzetti F, Carotti A, Terenzi A, Castellino F, Bonifacio E, et al. Tregs prevent GVHD and promote immune reconstitution in HLA-haploidentical transplantation. Blood. 2011;117:3921–8. 21292771 10.1182/blood-2010-10-311894

[16] Gaidot A, Landau DA, Martin GH, Bonduelle O, Grinberg-Bleyer Y, Matheoud D, et al. Immune reconstitution is preserved in hematopoietic stem cell transplantation coadministered with regulatory T cells for GVHD prevention. Blood. 2011;117:2975–83. 21193693 10.1182/blood-2010-08-299974

[17] Zhou G, Zhan Q, Huang L, Dou X, Cui J, Xiang L, et al. The dynamics of B-cell reconstitution post allogeneic hematopoietic stem cell transplantation: A real-world study. J Intern Med. 2024;295:634–50. 38439117 10.1111/joim.13776

[18] Jiang P, Yu F, Xu X, Cai Y, Yang J, Tong Y, et al. Impact of lymphocyte subsets of grafts on the outcome of Haploidentical peripheral blood stem cell transplantation. Cell Transplant. 2023;32:09636897231157054. 10.1177/09636897231157054 PMC10009013 36905323

[19] Zhang XH, Chen J, Han MZ, Huang H, Jiang EL, Jiang M, et al. The consensus from The Chinese Society of Hematology on indications, conditioning regimens and donor selection for allogeneic hematopoietic stem cell transplantation: 2021 update. J Hematol Oncol. 2021;14:145. 34526099 10.1186/s13045-021-01159-2 PMC8441240

[20] Bacigalupo A, Ballen K, Rizzo D, Giralt S, Lazarus H, Ho V, et al. Defining the Intensity of Conditioning Regimens: Working Definitions. Biol Blood Marrow Transplant. 2009;15:1628–33. 19896087 10.1016/j.bbmt.2009.07.004 PMC2861656

[21] Penack O, Marchetti M, Aljurf M, Arat M, Bonifazi F, Duarte RF, et al. Prophylaxis and management of graft-versus-host disease after stem-cell transplantation for haematological malignancies: updated consensus recommendations of the European Society for Blood and Marrow Transplantation. Lancet Haematol. 2024;11:e147–e59. 38184001 10.1016/S2352-3026(23)00342-3

[22] Wang Y, Liu Q-F, Lin R, Yang T, Xu Y-J, Mo X-D, et al. Optimizing antithymocyte globulin dosing in haploidentical hematopoietic cell transplantation: long-term follow-up of a multicenter, randomized controlled trial. Sci Bull. 2021;66:2498–505. 10.1016/j.scib.2021.06.002 36654209

[23] Döhner H, Wei AH, Appelbaum FR, Craddock C, DiNardo CD, Dombret H, et al. Diagnosis and management of AML in adults: 2022 recommendations from an international expert panel on behalf of the ELN. Blood. 2022;140:1345–77. 35797463 10.1182/blood.2022016867

[24] Greenberg PL, Tuechler H, Schanz J, Sanz G, Garcia-Manero G, Solé F, et al. Revised International Prognostic Scoring System for myelodysplastic syndromes. Blood. 2012;120:2454–65. 22740453 10.1182/blood-2012-03-420489 PMC4425443

[25] Shah B, Mattison RJ, Abboud R, Abdelmessieh P, Aldoss I, Burke PW, et al. Acute lymphoblastic leukemia, version 2.2024. J Natl Compr Canc Netw. 2024;22:563–76. 39413812 10.6004/jnccn.2024.0051

[26] Shipp MA, Harrington DP, Anderson JR, Armitage JO, Bonadonna G, Brittinger G, et al. A predictive model for aggressive non-Hodgkin’s lymphoma. N Engl J Med. 1993;329:987–94. 8141877 10.1056/NEJM199309303291402

[27] Przepiorka D, Weisdorf D, Martin P, Klingemann HG, Beatty P, Hows J, et al. Consensus conference on acute GVHD grading. Bone Marrow Transplant. 1995;15:825–8. 7581076

[28] Jagasia MH, Greinix HT, Arora M, Williams KM, Wolff D, Cowen EW, et al. National Institutes of Health consensus development project on criteria for clinical trials in chronic graft-versus-host disease: I. The 2014 Diagnosis and Staging Working Group report. Biol Blood Marrow Transplant. 2015;21:389–401. 25529383 10.1016/j.bbmt.2014.12.001 PMC4329079

[29] Imai K, Keele L, Tingley D. A general approach to causal mediation analysis. Psychol Methods. 2010;15:309–34. 20954780 10.1037/a0020761

[30] Lamer A, Laurent G, Pelayo S, El Amrani M, Chazard E, Marcilly R. Exploring patient path through sankey diagram: a proof of concept. Stud Health Technol Inf. 2020;270:218–22. 10.3233/SHTI200154 32570378

[31] Storek J. B-cell immunity after allogeneic hematopoietic cell transplantation. Cytotherapy. 2002;4:423–4. 12473209 10.1080/146532402320776026

[32] Kim DH, Sohn SK, Won DI, Lee NY, Suh JS, Lee KB. Rapid helper T-cell recovery above 200 ×106/l at 3 months correlates to successful transplant outcomes after allogeneic stem cell transplantation. Bone Marrow Transplant. 2006;37:1119–28. 16699530 10.1038/sj.bmt.1705381

[33] Bejanyan N, Brunstein CG, Cao Q, Lazaryan A, Luo X, Curtsinger J, et al. Delayed immune reconstitution after allogeneic transplantation increases the risks of mortality and chronic GVHD. Blood Adv. 2018;2:909–22. 29678809 10.1182/bloodadvances.2017014464 PMC5916001

[34] Velaga S, Ukena SN, Hoepting M, Ivanyi P, Borchers S, Mischak-Weissinger E-M, et al. Reconstitution and phenotype of Tregs in CMV reactivating patients following allogeneic hematopoietic stem cell transplantation. Immunol Invest. 2013;42:18–35. 23083129 10.3109/08820139.2012.719563

[35] Pastore D, Delia M, Mestice A, Perrone T, Carluccio P, Gaudio F, et al. Recovery of CMV-specific CD8+ T cells and Tregs after allogeneic peripheral blood stem cell transplantation. Biol Blood Marrow Transplant. 2011;17:550–7. 20457268 10.1016/j.bbmt.2010.04.011

[36] Almanan M, Raynor J, Sholl A, Wang M, Chougnet C, Cardin RD, et al. Tissue-specific control of latent CMV reactivation by regulatory T cells. PLoS Pathog. 2017;13:8. 10.1371/journal.ppat.1006507 PMC5552023 28796839

[37] Tian DM, Wang Y, Zhang XH, Liu KY, Huang XJ, Chang YJ. Rapid Recovery of CD3+ CD8+ T cells on day 90 predicts superior survival after unmanipulated haploidentical blood and marrow transplantation. PLoS ONE. 2016;11:e0156777. 10.1371/journal.pone.0156777 PMC4898737 27276058

[38] Yakoub-Agha I, Saule P, Magro L, Cracco P, Duhamel A, Coiteux V, et al. Immune reconstitution following myeloablative allogeneic hematopoietic stem cell transplantation: the impact of expanding CD28negative CD8 + T cells on relapse. Biol Blood Marrow Transplant. 2009;15:496–504. 19285638 10.1016/j.bbmt.2008.11.038

[39] Pei X-Y, Huang X-J. The role of immune reconstitution in relapse after allogeneic hematopoietic stem cell transplantation. Expert Rev Clin Immunol. 2024;20:513–24. 38599237 10.1080/1744666X.2023.2299728

[40] Ando T, Tachibana T, Tanaka M, Suzuki T, Ishiyama Y, Koyama S, et al. Impact of graft sources on immune reconstitution and survival outcomes following allogeneic stem cell transplantation. Blood Adv. 2020;4:408–19. 31990335 10.1182/bloodadvances.2019001021 PMC6988395

[41] van der Maas NG, Berghuis D, van der Burg M, Lankester AC. B cell reconstitution and influencing factors after hematopoietic stem cell transplantation in children. Front Immunol. 2019;10:782. 10.3389/fimmu.2019.00782 PMC6473193 31031769

[42] Tamaki M, Akahoshi Y, Inamoto Y, Morita K, Uchida N, Doki N, et al. Associations between acute and chronic graft-versus-host disease. Blood Adv. 2024;8:4250–61. 38985337 10.1182/bloodadvances.2024013442 PMC11372601

[43] Atkinson K, Horowitz MM, Gale RP, van Bekkum DW, Gluckman E, Good RA, et al. Risk factors for chronic graft-versus-host disease after HLA-identical sibling bone marrow transplantation. Blood. 1990;75:2459–64. 2350582

[44] Herzog S, Weisdorf DJ, Shanley R, Rayes A, Holtan SG, Young J-A, et al. Chronic GVHD after steroid-sensitive, -dependent, and -refractory acute GVHD: incidence and clinical outcomes. Blood Adv. 2023;7:3644–50. 37036949 10.1182/bloodadvances.2022009505 PMC10365934

[45] Franco LM, Gadkari M, Howe KN, Sun J, Kardava L, Kumar P, et al. Immune regulation by glucocorticoids can be linked to cell type-dependent transcriptional responses. J Exp Med. 2019;216:384–406. 30674564 10.1084/jem.20180595 PMC6363437

[46] Park KH, Ryu JH, Bae H, Yun S, Jang JH, Han K, et al. Delayed NK cell reconstitution and reduced NK activity increased the risks of CMV disease in allogeneic-hematopoietic stem cell transplantation. Int J Mol Sci. 2020;21:3663. 10.3390/ijms21103663 PMC7279475 32455959

[47] Pical-Izard C, Crocchiolo R, Granjeaud S, Kochbati E, Just-Landi S, Chabannon C, et al. Reconstitution of natural killer cells in HLA-matched HSCT after reduced-intensity conditioning: impact on clinical outcome. Biol Blood Marrow Transplant. 2015;21:429–39. 25579888 10.1016/j.bbmt.2014.11.681

[48] Russo A, Oliveira G, Berglund S, Greco R, Gambacorta V, Cieri N, et al. NK cell recovery after haploidentical HSCT with posttransplant cyclophosphamide: dynamics and clinical implications. Blood. 2018;131:247–62. 28986344 10.1182/blood-2017-05-780668 PMC5757695

[49] Peled A, Nagler A. NK cell destiny after haploSCT with PT-Cy. Blood. 2018;131:161–2. 29326351 10.1182/blood-2017-10-811117 PMC5757679

[50] Zhao C, Bartock M, Jia B, Shah N, Claxton DF, Wirk B, et al. Post-transplant cyclophosphamide alters immune signatures and leads to impaired T cell reconstitution in allogeneic hematopoietic stem cell transplant. J Hematol Oncol. 2022;15:64. 10.1186/s13045-022-01287-3 PMC9118756 35590334

[51] Massoud R, Gagelmann N, Fritzsche-Friedland U, Zeck G, Heidenreich S, Wolschke C, et al. Comparison of immune reconstitution between anti-T-lymphocyte globulin and posttransplant cyclophosphamide as acute graft-versus-host disease prophylaxis in allogeneic myeloablative peripheral blood stem cell transplantation. Haematologica. 2022;107:857–67. 33832208 10.3324/haematol.2020.271445 PMC8968885

