# Supplementary material for: Lymphocyte subset reconstitution and clinical outcomes following haploidentical hematopoietic stem cell transplantation
Source: Br J Cancer. 2026 Mar 2;134(9):1289–99. doi: 10.1038/s41416-026-03345-w (PMC13079791; doi:10.1038/s41416-026-03345-w)
Supplement: Supplementary file 1 — Supplementary material [file 41416_2026_3345_MOESM1_ESM.pdf]

Table S1. Univariate analysis of lymphocyte subset reconstitution counts at seven time points within one year post-transplant for their association with OS.

Cell subset (high vs low)	1 month		2 month		3 month		4 month		6 month		9 month		12 month	
	HR (95% CI)	P	HR (95% CI)	P	HR (95% CI)	P	HR (95% CI)	P	HR (95% CI)	P	HR (95% CI)	P	HR (95% CI)	P
NK cell	0.87 (0.49–1.57)	0.654	0.62 (0.38–1.04)	0.068	0.81 (0.52–1.28)	0.371	1.04 (0.61–1.76)	0.887	0.81 (0.48–1.38)	0.446	**0.38 (0.17–0.82)**	**0.014**	0.52 (0.26–1.06)	0.074
B cell	0.85 (0.47–1.52)	0.577	0.70 (0.42–1.16)	0.164	0.75 (0.48–1.19)	0.229	**0.50 (0.29–0.88)**	**0.016**	**0.48 (0.28–0.84)**	**0.011**	0.72 (0.35–1.48)	0.377	0.61 (0.31–1.23)	0.171
CD4+ T cell	0.58 (0.32–1.06)	0.075	0.96 (0.58–1.57)	0.861	1.06 (0.67–1.66)	0.817	**0.55 (0.32–0.95)**	**0.032**	0.69 (0.40–1.18)	0.174	0.56 (0.27–1.15)	0.115	0.65 (0.33–1.30)	0.228
Naive CD4+ T cell	0.74 (0.40–1.36)	0.333	0.70 (0.42–1.17)	0.171	0.81 (0.51–1.28)	0.365	0.59 (0.35–1.02)	0.06	0.63 (0.37–1.08)	0.095	0.49 (0.23–1.02)	0.056	0.77 (0.39–1.53)	0.454
Memory CD4+ T cell	0.59 (0.32–1.10)	0.096	1.01 (0.61–1.67)	0.97	1.05 (0.66–1.65)	0.841	**0.56 (0.33–0.97)**	**0.039**	0.74 (0.44–1.26)	0.271	0.50 (0.24–1.05)	0.067	0.70 (0.35–1.39)	0.306
Treg	**0.41 (0.20–0.84)**	**0.014**	**0.55 (0.31–0.98)**	**0.043**	0.95 (0.58–1.57)	0.852	**0.52 (0.28–0.99)**	**0.046**	1.05 (0.59–1.88)	0.863	0.75 (0.35–1.60)	0.454	0.55 (0.26–1.17)	0.122
CD8+ T cell	0.57 (0.31–1.04)	0.065	0.81 (0.49–1.34)	0.416	0.97 (0.62–1.53)	0.901	0.66 (0.39–1.13)	0.132	0.61 (0.35–1.05)	0.073	**0.36 (0.17–0.79)**	**0.011**	0.76 (0.38–1.51)	0.429
Naive CD8+ T cell	0.55 (0.29–1.02)	0.058	0.81 (0.49–1.35)	0.425	0.80 (0.51–1.27)	0.355	**0.48 (0.28–0.84)**	**0.01**	1.12 (0.66–1.90)	0.686	**0.35 (0.16–0.77)**	**0.009**	1.37 (0.68–2.76)	0.379
Memory CD8+ T cell	0.59 (0.32–1.10)	0.096	0.77 (0.46–1.28)	0.306	0.98 (0.62–1.54)	0.918	1.06 (0.63–1.80)	0.822	0.67 (0.39–1.15)	0.145	0.61 (0.30–1.26)	0.183	0.73 (0.37–1.46)	0.381
CD3+CD69+ T cell	0.60 (0.32–1.11)	0.104	0.83 (0.50–1.37)	0.466	0.92 (0.58–1.46)	0.722	0.96 (0.57–1.63)	0.879	0.94 (0.55–1.59)	0.81	0.64 (0.31–1.31)	0.22	0.75 (0.38–1.50)	0.419
CD3+HLA-DR+ T cell	0.59 (0.32–1.10)	0.099	0.75 (0.45–1.24)	0.267	0.87 (0.55–1.37)	0.541	0.95 (0.56–1.61)	0.836	0.63 (0.37–1.08)	0.093	**0.38 (0.17–0.82)**	**0.014**	0.54 (0.27–1.10)	0.09

Table S2. Univariate analysis of lymphocyte subset reconstitution counts at seven time points within one year post-transplant for their association with TRM.

Cell subset (high vs low)	1 month HR (95% CI)	1 month P	2 month HR (95% CI)	2 month P	3 month HR (95% CI)	3 month P	4 month HR (95% CI)	4 month P	6 month HR (95% CI)	6 month P	9 month HR (95% CI)	9 month P	12 month HR (95% CI)	12 month P
NK cell	1.01 (0.45–2.25)	0.982	**0.34 (0.15–0.76)**	**0.009**	0.50 (0.24–1.04)	0.065	1.00 (0.44–2.32)	0.993	1.06 (0.50–2.27)	0.871	0.80 (0.27–2.39)	0.691	0.42 (0.13–1.35)	0.144
B cell	0.58 (0.25–1.32)	0.193	**0.41 (0.19–0.90)**	**0.026**	**0.29 (0.12–0.67)**	**0.004**	**0.22 (0.07–0.64)**	**0.006**	**0.21 (0.08–0.56)**	**0.002**	**0.18 (0.04–0.82)**	**0.026**	0.28 (0.08–1.03)	0.056
CD4+ T cell	0.53 (0.23–1.22)	0.134	0.56 (0.27–1.17)	0.124	0.65 (0.32–1.31)	0.229	**0.33 (0.13–0.85)**	**0.022**	0.62 (0.29–1.34)	0.223	0.75 (0.25–2.23)	0.602	0.56 (0.18–1.72)	0.311
Naive CD4+ T cell	1.08 (0.47–2.51)	0.854	0.50 (0.23–1.09)	0.08	1.13 (0.56–2.29)	0.738	0.41 (0.17–1.01)	0.051	0.62 (0.29–1.35)	0.228	**0.27 (0.07–0.97)**	**0.044**	0.28 (0.08–1.00)	0.05
Memory CD4+ T cell	0.62 (0.26–1.44)	0.264	0.60 (0.28–1.27)	0.178	0.61 (0.29–1.25)	0.177	**0.34 (0.13–0.88)**	**0.026**	0.71 (0.33–1.53)	0.388	1.05 (0.35–3.12)	0.935	0.81 (0.27–2.42)	0.711
Treg	**0.26 (0.09–0.77)**	**0.016**	**0.18 (0.06–0.52)**	**0.002**	0.74 (0.33–1.67)	0.468	**0.33 (0.13–0.85)**	**0.022**	0.57 (0.22–1.44)	0.233	0.99 (0.29–3.41)	0.985	0.42 (0.11–1.63)	0.21
CD8+ T cell	0.52 (0.23–1.20)	0.124	0.51 (0.24–1.08)	0.08	**0.41 (0.19–0.86)**	**0.018**	0.62 (0.27–1.46)	0.275	0.84 (0.39–1.78)	0.644	0.39 (0.12–1.28)	0.12	1.08 (0.36–3.21)	0.896
Naive CD8+ T cell	**0.39 (0.16–0.96)**	**0.042**	0.50 (0.23–1.08)	0.076	**0.41 (0.19–0.87)**	**0.02**	**0.37 (0.15–0.92)**	**0.031**	1.33 (0.61–2.88)	0.469	**0.16 (0.04–0.72)**	**0.017**	1.46 (0.47–4.47)	0.511
Memory CD8+ T cell	0.50 (0.21–1.21)	0.124	0.58 (0.27–1.24)	0.159	0.51 (0.24–1.06)	0.072	1.26 (0.54–2.92)	0.591	0.73 (0.34–1.56)	0.419	1.12 (0.37–3.32)	0.845	0.62 (0.20–1.91)	0.409
CD3+CD69+ T cell	0.99 (0.43–2.28)	0.981	1.01 (0.49–2.09)	0.979	1.24 (0.60–2.57)	0.558	**2.63 (1.03–6.74)**	**0.044**	1.37 (0.64–2.97)	0.418	1.18 (0.39–3.50)	0.771	2.24 (0.69–7.28)	0.18
CD3+HLA-DR+ T cell	0.67 (0.29–1.56)	0.354	0.50 (0.23–1.07)	0.075	**0.40 (0.19–0.88)**	**0.022**	1.19 (0.51–2.75)	0.685	0.55 (0.25–1.20)	0.132	0.41 (0.13–1.33)	0.138	0.42 (0.13–1.36)	0.148

Table S3. Univariate analysis of lymphocyte subset reconstitution counts at seven time points within one year post-transplant for their association with RR.

Cell subset (high vs low)	1 month		2 month		3 month		4 month		6 month		9 month		12 month	
	HR (95% CI)	P	HR (95% CI)	P	HR (95% CI)	P	HR (95% CI)	P	HR (95% CI)	P	HR (95% CI)	P	HR (95% CI)	P
NK cell	1.08 (0.53–2.18)	0.84	1.08 (0.63–1.87)	0.779	1.41 (0.84–2.37)	0.198	1.09 (0.63–1.91)	0.75	0.88 (0.50–1.57)	0.675	0.60 (0.30–1.20)	0.148	0.88 (0.46–1.69)	0.694
B cell	1.20 (0.59–2.44)	0.61	1.29 (0.74–2.22)	0.37	1.49 (0.88–2.51)	0.137	0.95 (0.54–1.65)	0.849	0.91 (0.51–1.61)	0.746	1.11 (0.56–2.17)	0.771	0.67 (0.34–1.30)	0.235
CD4+ T cell	1.14 (0.56–2.31)	0.725	0.99 (0.58–1.71)	0.985	1.27 (0.76–2.13)	0.362	0.87 (0.50–1.51)	0.614	0.69 (0.38–1.22)	0.2	**0.42 (0.20–0.86)**	**0.018**	0.74 (0.38–1.42)	0.363
Naive CD4+ T cell	**0.45 (0.21–0.95)**	**0.037**	0.92 (0.53–1.58)	0.749	0.62 (0.37–1.04)	0.068	0.80 (0.46–1.39)	0.427	0.73 (0.41–1.30)	0.291	0.72 (0.36–1.41)	0.333	1.22 (0.63–2.36)	0.551
Memory CD4+ T cell	1.08 (0.52–2.21)	0.842	1.02 (0.59–1.75)	0.955	1.29 (0.77–2.16)	0.333	0.88 (0.50–1.53)	0.644	0.63 (0.35–1.13)	0.12	**0.32 (0.15–0.68)**	**0.003**	**0.46 (0.23–0.92)**	**0.028**
Treg	0.73 (0.34–1.56)	0.418	1.19 (0.65–2.17)	0.579	0.91 (0.53–1.57)	0.736	0.71 (0.38–1.31)	0.271	1.00 (0.55–1.82)	0.993	0.96 (0.48–1.91)	0.897	0.93 (0.48–1.83)	0.842
CD8+ T cell	0.87 (0.43–1.75)	0.69	1.01 (0.59–1.75)	0.967	1.49 (0.88–2.51)	0.137	0.76 (0.43–1.32)	0.326	0.59 (0.33–1.07)	0.081	**0.43 (0.21–0.88)**	**0.022**	**0.38 (0.19–0.78)**	**0.008**
Naive CD8+ T cell	0.87 (0.43–1.79)	0.711	0.92 (0.53–1.60)	0.775	1.02 (0.61–1.70)	0.945	**0.42 (0.23–0.75)**	**0.004**	0.71 (0.40–1.26)	0.24	**0.48 (0.24–0.97)**	**0.041**	0.63 (0.33–1.23)	0.177
Memory CD8+ T cell	0.94 (0.46–1.93)	0.875	0.95 (0.55–1.64)	0.849	1.41 (0.84–2.37)	0.196	1.12 (0.64–1.95)	0.69	0.86 (0.49–1.53)	0.609	0.61 (0.31–1.22)	0.165	0.67 (0.34–1.30)	0.233
CD3+CD69+ T cell	0.48 (0.22–1.02)	0.055	0.77 (0.44–1.33)	0.344	0.65 (0.39–1.10)	0.108	0.72 (0.41–1.27)	0.258	0.61 (0.34–1.09)	0.097	**0.30 (0.13–0.65)**	**0.003**	**0.37 (0.18–0.77)**	**0.008**
CD3+HLA-DR+ T cell	0.87 (0.43–1.79)	0.711	0.94 (0.55–1.62)	0.825	1.44 (0.86–2.42)	0.167	0.85 (0.49–1.49)	0.574	0.65 (0.36–1.16)	0.144	0.68 (0.34–1.35)	0.272	0.59 (0.30–1.15)	0.121

Table S4. Multivariable analysis of OS according to lymphocyte subset reconstitution status assessed at 1 month post-transplant.

Variables	Multivariate analysis	
	HR (95% CI)	P
Risk score (1=low, 2=intermediate, 3=high)	3.71 (1.31–10.5)	0.013
Disease status (NR vs CR)	2.08 (0.93–4.65)	0.071
Conditioning regimens (RIC vs MAC)	0.77 (0.29–2.02)	0.591
ECOG ≥2	2.05 (0.57–7.38)	0.275
HCT-CI ≥3	7.31 (2.73–19.6)	<0.001
Treg (High vs Low)	0.36 (0.17–0.75)	0.009

Table S5. Multivariable analysis of TRM according to lymphocyte subset reconstitution status assessed at 1 month post-transplant.

	Subset (high vs low)		Patient–donor sex (Female to male vs others)		Patient age (per year)		Disease status (NR vs CR)		Conditioning regimens (RIC vs MAC)		CMV reactivation (yes vs no)	
Cell subset	HR(95% CI)	P	HR(95% CI)	P	HR(95% CI)	P	HR(95% CI)	P	HR(95% CI)	P	HR(95% CI)	P
Treg	0.2 (0.09–0.82)	0.021	2.38 (0.89–6.40)	0.085	1.00 (0.96–1.05)	0.946	3.53 (1.36–9.14)	0.009	2.02 (0.47–8.70)	0.345	1.57 (0.60–4.07)	0.354
Naive CD8+ T cell	0.34 (0.13–0.87)	0.025	2.61 (1.06–6.40)	0.036	1.03 (0.99–1.08)	0.164	3.00 (1.25–7.24)	0.014	1.60 (0.43–5.97)	0.48	1.85 (0.78–4.38)	0.16

Table S6. Multivariable analysis of RR according to lymphocyte subset reconstitution status assessed at 1 month post-transplant.

Variables	Multivariate analysis	
	HR (95% CI)	P
ECOG ≥2	7.46 (2.40–23.2)	<0.001
Naive CD4+ T cell (High vs Low)	0.35 (0.16–0.79)	0.011

Table S7. Multivariable analysis of OS according to lymphocyte subset reconstitution status assessed at 2 months post-transplant.

Variables	Multivariate analysis	
	HR (95% CI)	P
Patient age (per year)	1.01 (0.99 – 1.04)	0.376
Disease status (NR vs CR)	2.13 (1.14 – 3.97)	0.018
Conditioning regimens (RIC vs MAC)	1.09 (0.48 – 2.49)	0.840
ECOG ≥2	1.48 (0.44 – 5.01)	0.526
Treg (High vs Low)	0.54 (0.30 – 0.97)	0.037

Table S8. Multivariable analysis of TRM according to lymphocyte subset reconstitution status assessed at 2 months post-transplant.

	Subset (high vs low)		Patient age (per year)		Disease status (NR vs CR)		Conditioning regimens (RIC vs MAC)		CMV reactivation (yes vs no)	
Cell subset	HR(95% CI)	P	HR(95% CI)	P	HR(95% CI)	P	HR(95% CI)	P	HR(95% CI)	P
Treg	0.20 (0.07–0.60)	0.004	1.03 (0.99–1.07)	0.095	2.52 (1.07–5.91)	0.034	0.956 (0.31–2.99)	0.938	2.41 (1.06–5.47)	0.038
NK cell	0.35 (0.16–0.80)	0.012	1.03 (0.99–1.06)	0.138	1.83 (0.85–3.98)	0.125	1.21 (0.44–3.31)	0.715	2.36 (1.14–4.88)	0.021
B cell	0.40 (0.18–0.89)	0.025	1.03 (1.00–1.07)	0.091	1.72 (0.80–3.73)	0.168	1.17 (0.44–3.16)	0.751	2.34 (1.13–4.86)	0.023

Table S9. Multivariable analysis of TRM according to lymphocyte subset reconstitution status assessed at 3 months post-transplant.

	Subset (high vs low)		Systemic steroid use		Patient age (per year)		CMV reactivation (yes vs no)		aGVHD grade 2–4	
Cell subset	HR(95% CI)	P	HR(95% CI)	P	HR(95% CI)	P	HR(95% CI)	P	HR(95% CI)	P
CD8+ T cell	0.47 (0.22–1.01)	0.053	1.36 (0.59–3.16)	0.474	1.03 (1.01–1.06)	0.008	2.37 (1.14–4.94)	0.021	2.75 (1.11–6.82)	0.03
B cell	0.38 (0.14–0.99)	0.048	0.90 (0.36–2.24)	0.826	1.04 (1.01–1.06)	0.003	2.05 (0.98–4.28)	0.056	2.80 (1.11–7.05)	0.029
CD3+HLA-DR+ T cell	0.57 (0.25–1.27)	0.169	1.14 (0.47–2.74)	0.774	1.03 (1.01–1.06)	0.01	2.42 (1.13–5.16)	0.023	2.97 (1.16–7.61)	0.023
Naive CD8+ T cell	0.40 (0.18–0.87)	0.02	1.41 (0.59–3.36)	0.443	1.03 (1.01–1.06)	0.011	2.53 (1.20–5.33)	0.015	3.25 (1.29–8.22)	0.013

Table S10. Multivariable analysis of OS according to lymphocyte subset reconstitution status assessed at 4 months post-transplant.

	Subset (high vs low)		Disease status (NR vs CR)		ECOG ≥2	
Cell subset	HR(95% CI)	P	HR(95% CI)	P	HR(95% CI)	P
Treg	0.45 (0.23–0.87)	0.016	1.52 (0.74–3.13)	0.245	5.00 (1.77–14.1)	0.002
CD4+ T cell	0.53 (0.31–0.89)	0.021	2.04 (1.14–3.66)	0.015	3.09 (1.32–7.22)	0.009
B cell	0.43 (0.24–0.76)	0.004	1.89 (1.06–3.36)	0.03	4.41 (1.83–10.7)	0.001
CD3+CD69+ T cell	0.83 (0.47–1.47)	0.505	1.94 (1.10–3.42)	0.023	3.42 (1.42–8.24)	0.006
Memory CD4+ T cell	0.54 (0.31–0.92)	0.024	2.04 (1.16–3.58)	0.015	3.09 (1.31–7.29)	0.009
Naive CD8+ T cell	0.47 (0.27–0.80)	0.008	2.03 (1.14–3.63)	0.016	3.00 (1.27–7.12)	0.012

Table S11. Multivariable analysis of TRM according to lymphocyte subset reconstitution status assessed at 4 months post-transplant.

Cell subset	Subset (high vs low)		Patient–donor sex (Female to male vs others)		Donor gender (female vs male)		CMV reactivation (yes vs no)	
	HR(95% CI)	P	HR(95% CI)	P	HR(95% CI)	P	HR(95% CI)	P
Treg	0.24 (0.07–0.81)	0.028	1.12 (0.21–6.02)	0.894	1.69 (0.35–8.18)	0.515	2.80 (0.97–8.08)	0.056
CD4+ T cell	0.42 (0.17–1.04)	0.075	2.09 (0.53–8.28)	0.35	1.38 (0.32–6.01)	0.677	2.78 (1.15–6.75)	0.023
Memory CD4+ T cell	0.40 (0.16–1.00)	0.058	2.10 (0.49–9.05)	0.345	1.43 (0.34–6.05)	0.644	2.86 (1.18–6.90)	0.019
B cell	0.28 (0.10–0.80)	0.024	1.83 (0.37–8.99)	0.444	1.53 (0.35–6.63)	0.586	2.57 (1.07–6.15)	0.036
CD3+CD69+ T cell	2.40 (0.91–6.35)	0.075	1.48 (0.31–6.92)	0.627	1.82 (0.38–8.67)	0.446	2.83 (1.16–6.89)	0.021
Naive CD8+ T cell	0.43 (0.17–1.07)	0.067	1.96 (0.43–8.86)	0.393	1.30 (0.30–5.71)	0.734	3.10 (1.28–7.50	0.01

Table S12. Multivariable analysis of RR according to lymphocyte subset reconstitution status assessed at 4 months post-transplant.

Variables	Multivariate analysis	
	HR (95% CI)	P
Disease status (NR vs CR)	1.37 (0.73–2.58)	0.333
CD3+ T cell dose in graft	0.63 (0.48–0.84)	0.001
CMV reactivation (yes vs no)	0.43 (0.21–0.87)	0.019
ECOG ≥2	4.57 (1.61–13.0)	0.004
Naive CD8+ T cell (High vs Low)	0.43 (0.23–0.79)	0.007

Table S13. Multivariable analysis of OS according to lymphocyte subset reconstitution status assessed at 6 months post-transplant.

Variables	Multivariate analysis	
	HR (95% CI)	P
Risk score (1=low, 2=intermediate, 3=high)	0.94 (0.51–1.76)	0.856
Disease status (NR vs CR)	1.97 (1.08–3.58)	0.027
EBV reactivation	1.51 (0.85–2.68)	0.156
ECOG ≥2	2.22 (0.76–6.47)	0.146
aGVHD grade 2–4	1.90 (0.96–3.76)	0.064
B cells (High vs Low)	0.53 (0.30–0.95)	0.033

Table S14. Multivariable analysis of TRM according to lymphocyte subset reconstitution status assessed at 6 months post-transplant.

Variables	Multivariate analysis	
	HR (95% CI)	P value
Systemic steroid use	1.71 (0.57–5.17)	0.34
Patient age (per year)	1.04 (1.01–1.07)	0.009
Disease status (NR vs CR)	1.68 (0.76–3.72)	0.203
CMV reactivation (yes vs no)	1.72 (0.74–4.00)	0.207
aGVHD grade 2–4	3.25 (1.35–7.84)	0.009
B cells (High vs Low)	0.30 (0.11–0.80)	0.017

Table S15. Multivariable analysis of OS according to lymphocyte subset reconstitution status assessed at 9 months post-transplant.

Cell subset	Subset (high vs low)		ECOG ≥2	
	HR(95% CI)	P	HR(95% CI)	P
CD8+ T cell	0.39 (0.18–0.85)	0.019	6.17 (2.35–16.2)	<0.001
NK cell	0.43 (0.20–0.95)	0.035	5.75 (2.17–15.2)	<0.001
CD3+HLA-DR+ T cell	0.45 (0.20–1.01)	0.055	5.11 (1.89–13.8)	0.001
Naive CD8+ T cell	0.30 (0.14–0.66)	0.003	9.49 (3.54–25.5)	<0.001

Table S16. Multivariable analysis of TRM according to lymphocyte subset reconstitution status assessed at 9 months post-transplant.

Cell subset	Subset (high vs low)		CMV reactivation (yes vs no)	
	HR(95% CI)	P	HR(95% CI)	P
B cell	0.21 (0.05–0.90)	0.044	2.95 (0.90–9.70)	0.075
Naive CD8+ T cell	0.11 (0.02–0.50)	0.004	5.59 (1.69–18.5)	0.005

Table S17. Multivariable analysis of RR according to lymphocyte subset reconstitution status assessed at 9 months post-transplant.

	Subset (high vs low)		CD3+ T cell dose in graft		ECOG ≥2	
Cell subset	HR(95% CI)	P	HR(95% CI)	P	HR(95% CI)	P
CD4+ T cell (high)	0.44 (0.21–0.94)	0.029	0.66 (0.47–0.92)	0.017	4.40 (1.32–14.7)	0.015
CD8+ T cell (high)	0.48 (0.23–0.99)	0.049	0.65 (0.47–0.90)	0.013	3.08 (0.93–10.2)	0.068
CD3+CD69+ T cell	0.31 (0.15–0.64)	0.004	0.62 (0.44–0.88)	0.008	3.21 (0.98–10.5)	0.055
Memory CD4+ (CD45RO)	0.34 (0.17–0.74)	0.006	0.66 (0.47–0.92)	0.019	4.61 (1.29–16.4)	0.012
Naive CD8+ (CD45RA)	0.53 (0.26–1.09)	0.086	0.65 (0.46–0.91)	0.015	4.38 (1.33–14.5)	0.016

Table S18. Multivariable analysis of RR according to lymphocyte subset reconstitution status assessed at 9 months post-transplant.

	Subset (high vs low)		CD3+ T cell dose in graft	
Cell subset	HR(95% CI)	P	HR(95% CI)	P
CD8+ T cell	0.40 (0.20–0.81)	0.013	0.68 (0.52–0.89)	0.006
CD3+CD69+ T cell	0.34 (0.17–0.69)	0.003	0.64 (0.47–0.86)	0.002
Memory CD4+ T cell	0.48 (0.24–0.96)	0.039	0.67 (0.49–0.90)	0.005

Table S19. Univariate analysis of lymphocyte subset reconstitution counts (as continuous variables) at seven time points within one year post-transplant for their association with OS.

	1 month		2 month		3 month		4 month		6 month		9 month		12 month	
Cell Subset	HR (95% CI)	P	HR (95% CI)	P	HR (95% CI)	P	HR (95% CI)	P	HR (95% CI)	P	HR (95% CI)	P	HR (95% CI)	P
NK cell	0.96 (0.71–1.30)	0.808	0.94 (0.72–1.22)	0.623	0.98 (0.77–1.23)	0.839	0.97 (0.74–1.26)	0.799	0.89 (0.68–1.16)	0.389	**0.66 (0.45–0.96)**	**0.030**	0.70 (0.46–1.05)	0.084
B cell	0.85 (0.63–1.16)	0.311	0.78 (0.59–1.04)	0.087	0.83 (0.64–1.07)	0.144	**0.76 (0.58–1.00)**	**0.050**	**0.75 (0.57–0.99)**	**0.041**	0.81 (0.56–1.17)	0.257	0.73 (0.50–1.05)	0.092
CD4+ T cell	0.78 (0.57–1.06)	0.113	0.89 (0.68–1.16)	0.39	0.97 (0.77–1.23)	0.812	**0.67 (0.51–0.89)**	**0.006**	**0.87 (0.66–1.14)**	0.308	0.73 (0.51–1.06)	0.102	0.91 (0.64–1.29)	0.592
Naive CD4+ T cell	0.89 (0.66–1.20)	0.444	0.89 (0.68–1.17)	0.41	0.94 (0.74–1.19)	0.594	**0.69 (0.52–0.92)**	**0.011**	**0.70 (0.53–0.93)**	**0.013**	**0.62 (0.42–0.92)**	**0.017**	0.81 (0.56–1.17)	0.252
Memory CD4+ T cell	0.78 (0.58–1.06)	0.117	0.90 (0.69–1.17)	0.413	0.91 (0.72–1.15)	0.423	**0.67 (0.51–0.89)**	**0.005**	0.87 (0.67–1.14)	0.323	0.69 (0.48–1.01)	0.054	0.93 (0.65–1.31)	0.660
Treg	**0.67 (0.48–0.94)**	**0.021**	0.81 (0.60–1.09)	0.166	0.82 (0.63–1.05)	0.116	0.78 (0.59–1.03)	0.079	0.99 (0.75–1.29)	0.931	0.85 (0.59–1.22)	0.374	0.76 (0.52–1.11)	0.151
CD8+ T cell	**0.66 (0.48–0.91)**	**0.011**	0.82 (0.63–1.07)	0.141	0.96 (0.76–1.21)	0.701	0.77 (0.58–1.01)	0.060	0.86 (0.66–1.12)	0.263	**0.61 (0.42–0.90)**	**0.012**	0.82 (0.57–1.16)	0.262
Naive CD8+ T cell	0.74 (0.54–1.01)	0.061	0.95 (0.74–1.23)	0.702	0.92 (0.73–1.17)	0.508	**0.71 (0.53–0.94)**	**0.017**	1.07 (0.82–1.40)	0.605	**0.57 (0.38–0.85)**	**0.006**	0.95 (0.67–1.33)	0.746
Memory CD8+ T cell	**0.68 (0.50–0.94)**	**0.021**	0.84 (0.64–1.09)	0.183	0.98 (0.78–1.24)	0.892	0.89 (0.68–1.16)	0.392	0.82 (0.63–1.07)	0.147	**0.67 (0.46–0.97)**	**0.034**	0.78 (0.54–1.12)	0.185

CD3+CD69+ T cell	0.81 (0.60–1.10)	0.179	0.96 (0.74–1.23)	0.727	0.92 (0.73–1.17)	0.514	0.97 (0.75–1.27)	0.843	0.95 (0.73–1.23)	0.683	0.82 (0.57–1.18)	0.276	0.95 (0.67–1.35)	0.774
CD3+HLA-DR + T cell	**0.68 (0.49–0.94)**	**0.019**	**0.69 (0.51–0.95)**	**0.022**	0.86 (0.67–1.10)	0.230	1.02 (0.78–1.33)	0.876	0.76 (0.58–1.00)	0.053	**0.58 (0.39–0.87)**	**0.009**	0.79 (0.54–1.15)	0.216

Table S20. Univariate analysis of lymphocyte subset reconstitution counts (as continuous variables) at seven time points within one year post-transplant for their association with TRM.

	1 month		2 month		3 month		4 month		6 month		9 month		12 month	
Cell Subset	HR (95% CI)	P	HR (95% CI)	P	HR (95% CI)	P	HR (95% CI)	P	HR (95% CI)	P	HR (95% CI)	P	HR (95% CI)	P
NK cell	1.02 (0.68–1.54)	0.909	**0.59 (0.36–0.99)**	**0.046**	**0.60 (0.37–0.97)**	**0.036**	1.02 (0.67–1.55)	0.920	0.91 (0.62–1.33)	0.624	0.92 (0.53–1.58)	0.751	0.63 (0.32–1.26)	0.192
B cell	0.62 (0.38–1.01)	0.054	**0.49 (0.28–0.85)**	**0.011**	**0.45 (0.25–0.80)**	**0.007**	**0.52 (0.32–0.87)**	**0.012**	**0.49 (0.30–0.78)**	**0.003**	**0.40 (0.19–0.86)**	**0.019**	**0.31 (0.13–0.71)**	**0.006**
CD4+ T cell	0.73 (0.47–1.13)	0.159	**0.60 (0.37–0.96)**	**0.034**	**0.65 (0.43–0.99)**	**0.044**	**0.57 (0.35–0.91)**	**0.019**	0.85 (0.57–1.24)	0.392	0.90 (0.52–1.56)	0.701	0.84 (0.48–1.49)	0.559
Naive CD4+ T cell	1.09 (0.74–1.61)	0.668	0.71 (0.44–1.16)	0.17	0.93 (0.64–1.34)	0.683	**0.58 (0.35–0.94)**	**0.027**	0.68 (0.45–1.02)	0.063	**0.44 (0.22–0.90)**	**0.024**	**0.35 (0.13–0.95)**	**0.039**
Memory CD4+ T cell	0.79 (0.52–1.20)	0.274	**0.61 (0.38–0.98)**	**0.043**	**0.55 (0.36–0.86)**	**0.008**	**0.55 (0.34–0.88)**	**0.013**	0.87 (0.59–1.28)	0.484	0.93 (0.54–1.60)	0.788	1.05 (0.62–1.80)	0.847
Treg	**0.59 (0.36–0.96)**	**0.035**	**0.37 (0.18–0.76)**	**0.007**	0.68 (0.43–1.05)	0.084	**0.51 (0.30–0.87)**	**0.013**	0.77 (0.52–1.16)	0.212	0.95 (0.55–1.66)	0.866	0.55 (0.27–1.10)	0.089
CD8+ T cell	**0.61 (0.38–0.96)**	**0.033**	**0.61 (0.40–0.93)**	**0.022**	**0.63 (0.42–0.95)**	**0.026**	0.76 (0.49–1.18)	0.220	0.98 (0.67–1.43)	0.917	0.69 (0.39–1.22)	0.200	0.93 (0.54–1.61)	0.796

Naive CD8+ T cell	0.70 (0.45–1.09)	0.111	**0.57 (0.34–0.96)**	**0.036**	**0.63 (0.41–0.99)**	**0.044**	0.67 (0.43–1.06)	0.084	1.21 (0.82–1.78)	0.342	**0.42 (0.20–0.87)**	**0.019**	0.98 (0.57–1.68)	0.944
Memory CD8+ T cell	0.64 (0.41–1.01)	0.057	0.70 (0.47–1.06)	0.092	**0.64 (0.42–0.97)**	**0.038**	0.96 (0.63–1.46)	0.832	0.87 (0.59–1.27)	0.460	0.86 (0.50–1.50)	0.604	0.86 (0.49–1.50)	0.591
CD3+CD69+ T cell	0.92 (0.62–1.38)	0.699	0.89 (0.61–1.32)	0.573	0.83 (0.56–1.21)	0.330	**1.59 (1.01–2.50)**	**0.047**	1.22 (0.83–1.79)	0.304	1.06 (0.61–1.83)	0.831	1.53 (0.95–2.47)	0.082
CD3+HLA-DR+ T cell	0.65 (0.41–1.03)	0.067	**0.50 (0.29–0.88)**	**0.015**	**0.40 (0.22–0.71)**	**0.002**	1.14 (0.75–1.75)	0.541	0.70 (0.47–1.04)	0.078	0.57 (0.30–1.06)	0.077	0.62 (0.32–1.21)	0.162

Table S21. Univariate analysis of lymphocyte subset reconstitution counts (as continuous variables) at seven time points within one year post-transplant for their association with RR.

Cell Subset	1 month		2 month		3 month		4 month		6 month		9 month		12 month	
	HR (95% CI)	P	HR (95% CI)	P	HR (95% CI)	P	HR (95% CI)	P	HR (95% CI)	P	HR (95% CI)	P	HR (95% CI)	P
NK cell	1.14 (0.80–1.62)	0.48	1.13 (0.88–1.46)	0.332	1.21 (0.95–1.53)	0.121	0.98 (0.74–1.29)	0.876	0.99 (0.75–1.32)	0.965	0.81 (0.57–1.14)	0.226	0.82 (0.57–1.17)	0.268
B cell	1.10 (0.78–1.55)	0.585	1.08 (0.83–1.41)	0.551	1.07 (0.83–1.36)	0.613	1.04 (0.79–1.37)	0.790	0.99 (0.74–1.32)	0.960	1.01 (0.72–1.42)	0.944	0.90 (0.64–1.26)	0.525
CD4+ T cell	1.03 (0.73–1.47)	0.853	1.09 (0.84–1.42)	0.513	1.14 (0.89–1.46)	0.311	0.86 (0.65–1.13)	0.273	0.78 (0.58–1.04)	0.095	**0.64 (0.44–0.93)**	**0.019**	0.79 (0.56–1.12)	0.182
Naive CD4+ T cell	**0.66 (0.43–0.99)**	**0.047**	1.02 (0.78–1.32)	0.911	0.92 (0.70–1.21)	0.549	0.81 (0.61–1.07)	0.141	0.79 (0.59–1.06)	0.109	0.83 (0.59–1.17)	0.278	1.08 (0.79–1.48)	0.629
Memory CD4+ T cell	1.02 (0.72–1.45)	0.909	1.07 (0.82–1.39)	0.606	1.11 (0.87–1.43)	0.401	0.88 (0.66–1.16)	0.361	0.78 (0.58–1.05)	0.102	**0.60 (0.41–0.87)**	**0.007**	**0.67 (0.47–0.97)**	**0.035**

Treg	0.86 (0.59–1.24)	0.41	1.06 (0.81–1.38)	0.685	0.91 (0.70–1.19)	0.499	0.88 (0.66–1.17)	0.383	0.94 (0.70–1.26)	0.683	0.96 (0.68–1.35)	0.820	1.07 (0.78–1.48)	0.679
CD8+ T cell	0.93 (0.65–1.33)	0.688	0.91 (0.69–1.20)	0.488	1.16 (0.90–1.49)	0.248	0.80 (0.60–1.06)	0.126	0.79 (0.59–1.07)	0.124	**0.62 (0.43–0.89)**	**0.010**	**0.63 (0.44–0.90)**	**0.012**
Naive CD8+ T cell	0.86 (0.60–1.24)	0.427	1.06 (0.82–1.38)	0.633	1.03 (0.80–1.32)	0.812	**0.61 (0.45–0.83)**	**0.002**	0.79 (0.59–1.06)	0.119	**0.66 (0.46–0.95)**	**0.027**	**0.68 (0.47–0.98)**	**0.039**
Memory CD8+ T cell	0.96 (0.68–1.37)	0.843	0.88 (0.66–1.16)	0.361	1.22 (0.96–1.56)	0.105	0.94 (0.71–1.25)	0.690	0.91 (0.68–1.21)	0.505	**0.69 (0.48–0.99)**	**0.042**	0.73 (0.52–1.04)	0.085
CD3+CD69+ T cell	0.80 (0.56–1.16)	0.237	1.06 (0.82–1.38)	0.655	0.94 (0.72–1.22)	0.628	0.83 (0.63–1.11)	0.207	0.76 (0.56–1.02)	0.069	**0.54 (0.36–0.82)**	**0.003**	**0.61 (0.40–0.94)**	**0.025**
CD3+HLA-DR + T cell	0.99 (0.70–1.41)	0.964	0.88 (0.66–1.17)	0.369	1.16 (0.91–1.47)	0.240	0.97 (0.74–1.29)	0.858	0.76 (0.57–1.03)	0.076	0.79 (0.56–1.12)	0.191	0.82 (0.58–1.16)	0.258

Table S22. Multivariate analysis of cumulative incidence of CMV reactivation according to Treg reconstitution status assessed at 1 month post-transplant.

Variables	Multivariate analysis	
	HR (95% CI)	P
Letermovir prophylaxis (yes vs no)	0.20 (0.09–0.45)	<0.001
Disease status (NR vs CR)	1.59 (0.81–3.10)	0.175
Treg (high vs low)	0.49 (0.26–0.94)	0.032

Table S23. Multivariate analysis of cumulative incidence of CMV reactivation according to Treg and NK cell reconstitution status assessed at 2 months post-transplant.

Cell subset	Subset (high vs low)		Letermovir prophylaxis (yes vs no)		Donor gender (female vs male)		CD3+ T cell dose in graft	
	HR(95% CI)	P	HR(95% CI)	P	HR(95% CI)	P	HR(95% CI)	P
Treg (high vs low)	0.22 (0.09-0.55)	0.001	0.37 (0.16-0.87)	0.022	0.47 (0.18-1.24)	0.125	0.77 (0.56-1.05)	0.094
NK cell (high vs low)	0.59 (0.23-1.53)	0.279	0.59 (0.2-1.78)	0.352	0.3 (0.12-0.78)	0.014	0.78 (0.59-1.03)	0.081

Table S24. Multivariable analysis of cumulative incidence of cGVHD according to B cell reconstitution status assessed at 2 months post-transplant.

Variables	Multivariate analysis	
	HR (95% CI)	P
Systemic steroid use	5.64 (2.46–12.9)	<0.001
B cell (high vs low)	1.08 (0.55–2.13)	0.828

Table S25. Multivariable analysis of cumulative incidence of cGVHD according to B cell reconstitution status assessed at 6 months post-transplant.

Variables	Multivariate analysis	
	HR (95% CI)	P
Systemic steroid use	6.17 (2.58–14.8)	<0.001
Disease status (NR vs CR)	1.43 (0.72–2.85)	0.308
CD3+ T cell dose in graft	1.20 (1.00–1.43)	0.048
ECOG ≥2	3.90 (1.11–13.7)	0.035
B cells (high vs low)	0.54 (0.28–1.03)	0.062

Table S26. Multivariable analysis of cumulative incidence of cGVHD according to B cell reconstitution status assessed at 9 months post-transplant.

Variables	Multivariate analysis	
	HR (95% CI)	P
Systemic steroid use	2.83 (1.00–8.04)	0.051
B cells (high vs low)	0.32 (0.10–0.99)	0.047

Figure S1. Impact of GVHD prophylaxis on lymphocyte subset reconstitution.

Panels A–G show box-and-whisker plots of absolute counts (cells/μL) for the indicated lymphocyte subsets at 1, 2, 3, 4, 6, 9, and 12 months after transplantation, respectively, stratified by GVHD prophylaxis regimen: ATG+PT-Cy, ATG alone, and PT-Cy alone. ATG, anti-thymocyte globulin; PTCY, posttransplant cyclophosphamide.

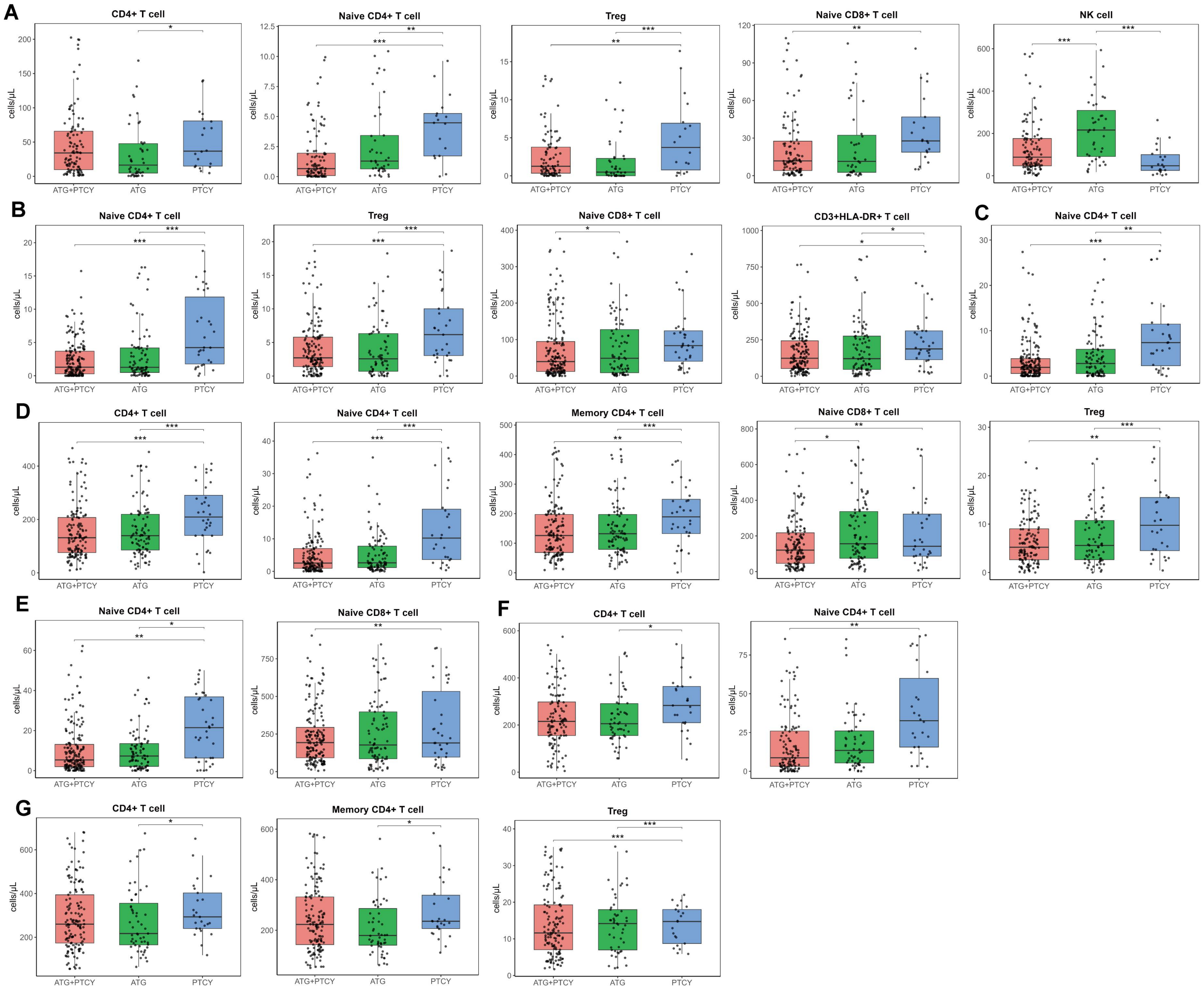
A
CD4+ T cell
Naive CD4+ T cell
Treg
Naive CD8+ T cell
NK cell
B
Naive CD4+ T cell
Treg
Naive CD8+ T cell
CD3+HLA-DR+ T cell
C
Naive CD4+ T cell
D
CD4+ T cell
Naive CD4+ T cell
Memory CD4+ T cell
Naive CD8+ T cell
Treg
E
Naive CD4+ T cell
Naive CD8+ T cell
F
CD4+ T cell
Naive CD4+ T cell
G
CD4+ T cell
Memory CD4+ T cell
Treg
cells/μL
ATG+PTCY
ATG
PTCY

Figure S2. Complete set of lymphocyte reconstitution–outcome curves moved from the main figure.

(A) OS: Kaplan–Meier curves for the remaining subset–timepoint combinations not shown in Figure 2 (see panel headers for the specific subset and month).(B) TRM: cumulative-incidence curves for the remaining subset–timepoint combinations.(C) RR: cumulative-incidence curves for the remaining subset–timepoint combinations.At each indicated month, subsets were dichotomized by the median absolute count (cells/μL). P values displayed in each panel are from multivariable Cox proportional hazards models. Curves are shown as Kaplan–Meier (OS) or cumulative incidence (TRM/RR) for visualization; OS panels include numbers at risk, and TRM/RR panels include cumulative numbers of events. Shaded areas represent 95% confidence intervals, and tick marks indicate censoring. OS, overall survival; TRM, transplant-related mortality; RR, relapse rate.

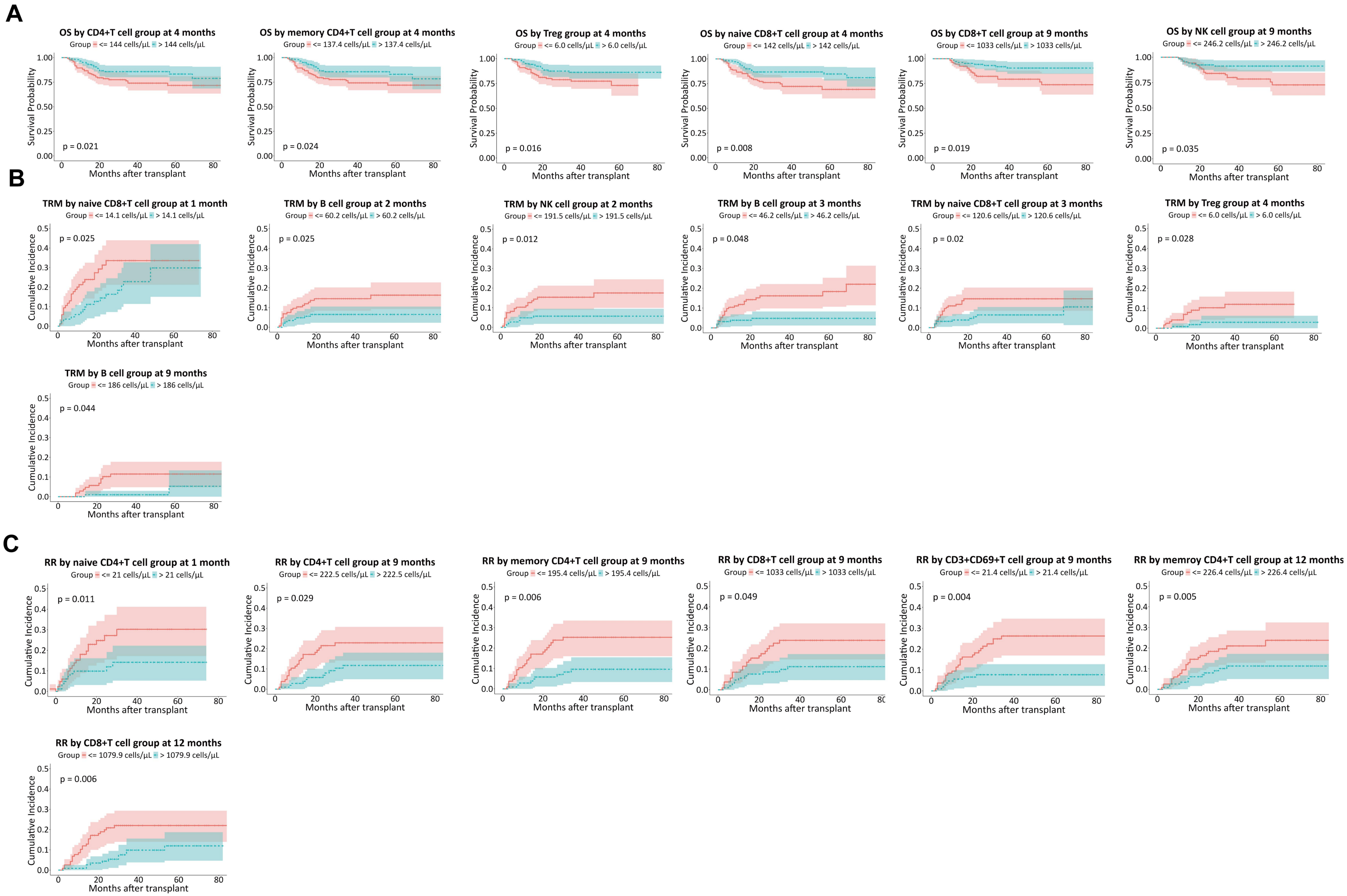
A
OS by CD4+T cell group at 4 months
Group <= 144 cells/μL > 144 cells/μL
p = 0.021
OS by memory CD4+T cell group at 4 months
Group <= 137.4 cells/μL > 137.4 cells/μL
p = 0.024
OS by Treg group at 4 months
Group <= 6.0 cells/μL > 6.0 cells/μL
p = 0.016
OS by naive CD8+T cell group at 4 months
Group <= 142 cells/μL > 142 cells/μL
p = 0.008
OS by CD8+T cell group at 9 months
Group <= 1033 cells/μL > 1033 cells/μL
p = 0.019
OS by NK cell group at 9 months
Group <= 246.2 cells/μL > 246.2 cells/μL
p = 0.035
Survival Probability
Months after transplant
B
TRM by naive CD8+T cell group at 1 month
Group <= 14.1 cells/μL > 14.1 cells/μL
p = 0.025
TRM by B cell group at 2 months
Group <= 60.2 cells/μL > 60.2 cells/μL
p = 0.025
TRM by NK cell group at 2 months
Group <= 191.5 cells/μL > 191.5 cells/μL
p = 0.012
TRM by B cell group at 3 months
Group <= 46.2 cells/μL > 46.2 cells/μL
p = 0.048
TRM by naive CD8+T cell group at 3 months
Group <= 120.6 cells/μL > 120.6 cells/μL
p = 0.02
TRM by Treg group at 4 months
Group <= 6.0 cells/μL > 6.0 cells/μL
p = 0.028
TRM by B cell group at 9 months
Group <= 186 cells/μL > 186 cells/μL
p = 0.044
Cumulative Incidence
Months after transplant
C
RR by naive CD4+T cell group at 1 month
Group <= 21 cells/μL > 21 cells/μL
p = 0.011
RR by CD4+T cell group at 9 months
Group <= 222.5 cells/μL > 222.5 cells/μL
p = 0.029
RR by memory CD4+T cell group at 9 months
Group <= 195.4 cells/μL > 195.4 cells/μL
p = 0.006
RR by CD8+T cell group at 9 months
Group <= 1033 cells/μL > 1033 cells/μL
p = 0.049
RR by CD3+CD69+T cell group at 9 months
Group <= 21.4 cells/μL > 21.4 cells/μL
p = 0.004
RR by memroy CD4+T cell group at 12 months
Group <= 226.4 cells/μL > 226.4 cells/μL
p = 0.005
RR by CD8+T cell group at 12 months
Group <= 1079.9 cells/μL > 1079.9 cells/μL
p = 0.006
Cumulative Incidence
Months after transplant
